# Neocornuside A–D, Four Novel Iridoid Glycosides from Fruits of *Cornus officinalis* and Their Antidiabetic Activity

**DOI:** 10.3390/molecules27154732

**Published:** 2022-07-24

**Authors:** Meng Yang, Zhiyou Hao, Xiaolan Wang, Shiqi Zhou, Denghui Zhu, Ying Yang, Junjun Wei, Meng Li, Xiaoke Zheng, Weisheng Feng

**Affiliations:** 1 School of Pharmacy, Henan University of Chinese Medicine, Zhengzhou 450046, China; ym199928@163.com (M.Y.); hzy@hactcm.edu.cn (Z.H.); 18337665752@163.com (S.Z.); z18737420720@163.com (D.Z.); yy412169@163.com (Y.Y.); myshf1234@163.com (J.W.); limeng31716@163.com (M.L.); 2 The Engineering and Technology Center for Chinese Medicine Development of Henan Province China, Zhengzhou 450046, China; wxl0325@hactcm.edu.cn; 3 Academy of Chinese Medical Sciences, Henan University of Chinese Medicine, Zhengzhou 450046, China

**Keywords:** *Cornus officinalis*, iridoid glycosides, structure elucidation, antidiabetic activity

## Abstract

Four previously undescribed iridoid glycosides neocornuside A–D (**1–4**), along with six known ones (**5–10**), were isolated from *Cornus officinalis* fruit. Their structures were elucidated by extensive spectroscopic (NMR, UV, IR, and MS) analysis and comparison with data reported in the literature. All isolates were assessed for their antidiabetic activity on the relative glucose consumption in insulin-induced insulin-resistant HepG2 cells. The results showed that compounds **1**, **3**, and **7** exhibited significant antidiabetic activities with EC_50_ values of 0.582, 1.275, and 0.742 μM, respectively. Moreover, compounds **1**, **3**, and **7** could improve the ability of 2-NBDG uptake of insulin-induced HepG2 cells.

## 1. Introduction

*Cornus officinalis* (Cornaceae), known as “Shanzhuyu” in Chinese, is a frequently used traditional Chinese medicine and widely distributed in eastern Asia, mainly in China, Korea, and Japan [1]. As a medicinal and food-homologous traditional Chinese medicine, previous phytochemical research demonstrated the occurrences of structurally diverse iridoids, flavonoids, lignans, tannins, terpenoids, and phenolic acids [2,3,4,5,6], with a wide range of pharmacological activities, including antidiabetic, anti-Alzheimer’s disease, anti-inflammatory, anti-tumor, hepatoprotective, neuroprotective, anti-oxidative, and anti-bacterial activities [7,8,9,10,11,12,13].

**Figure 1:**
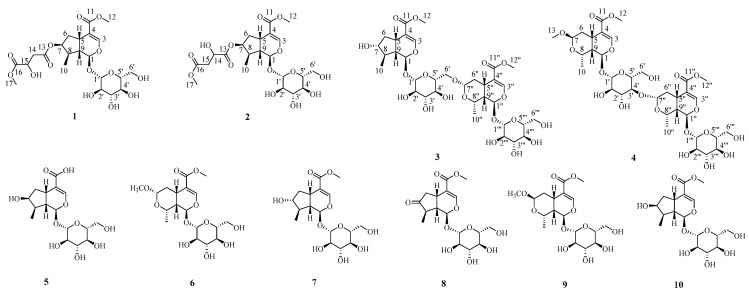
Chemical structures of compounds **1**–**10** isolated from the fruits of *Cornus officinalis*.

Diabetes mellitus (DM) is a major health problem for the people all over the world. In recent years, the global incidence of diabetes has increased rapidly due to many factors such as the improvement of living standards, changes in dietary structure, increasingly stressful rhythm of life, a less active and more sedentary lifestyle, and diabetes has become the third most serious chronic disease threatening human health after tumors and cardiovascular disease [14,15]. It has been linked to oxidative stress, which arises mainly through oxidation, oxidative degradation of glycated proteins, and nonenzymatic protein glycation [14]. Plant products and their derivatives have been widely accepted to possess many pharmacological activities, such as anti-inflammatory, antimicrobial, anticancer and antidiabetic activity. *Coptis chinensis* Franch (*Ranunculaceae*) polysaccharide (CCPW) can produce antidiabetic activity in rats with T2DM through its antioxidative effect, which is closely related to the JNK/IRS1/PI3K pathway [16]. Three polysaccharides were extracted from *Suillellus luridus* (Suilu.A, Suilu.C, and Suilu.S) which exhibited significant antidiabetic activity in diabetic mice induced by streptozotocin [17]. Moreover, the methanolic extract of *Geigeriaalata* has antidiabetic activity and it is suggested that this antidiabetic activity is due to enhanced insulin secretion, modulation of *β*-cell function, and improvement of antioxidant status [18]. Thus, traditional medicines have been proved to be a vital source of future drugs to prevent and treat many diseases, including diabetes mellitus. Iridoids are one of the major and characteristic ingredients of *Cornus officinalis*. The previous study proved the reasonableness of using iridoids isolated from *Cornus officinalis* to treat diabetes [15,19]. *C. officinalis* extracts and pure compounds could ameliorate diabetes-associated damages and complications. Oral administration of loganin and morroniside decreased fasting blood glucose levels in diabetes mellitus mice. Ursolic acid exhibited the highest reactive oxygen species scavenging activity and *α*-glucosidase inhibitory activity [7]. In addition, loganic acid (LA) exhibited antioxidant properties in relation to STZ-induced DM. It may indicate LA as one of the plant components in the development of new drugs that will treat metabolic and functional disorders in leukocytes under diabetes [20]. In recent years, there is growing interest in the utilization of natural products as potential therapeutic agents for treating DM. Therefore, in order to find natural products with antidiabetic activity from this plant, we systematically studied the fruits of *Cornus officinalis*. In our recent study, four new iridoid glycosides (**1**–**4**) and six known ones (**5**–**10**) were obtained from *Cornus officinalis* (Figure 1). In addition, the in vitro antidiabetic activity of the isolated compounds was evaluated.

## 2. Results and Discussion

### 2.1. Structure Elucidation

**Figure 2:**
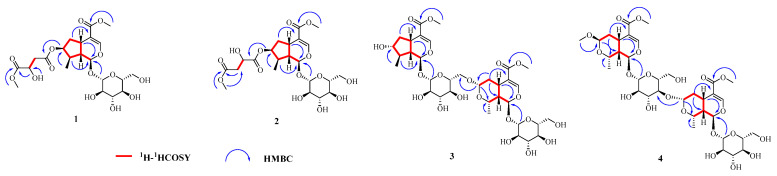
The key HMBC and ^1^H-^1^H COSY correlations of compounds **1**–**4.**

**Figure 3:**
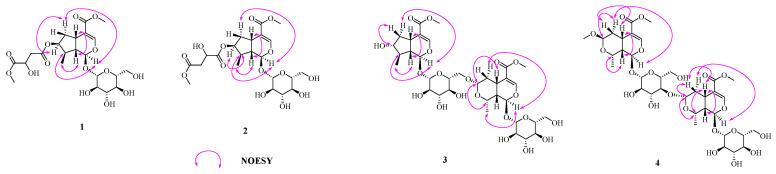
The key NOESY correlations of compounds **1**–**4.**

Compound **1** was assigned a molecular formula of C_22_H_32_O_14_, as determined from HRESIMS (*m/z*: 543.1689 [M + Na]^+^) and ^13^C NMR data (Table 1). The ^1^H NMR and ^13^C NMR spectrum displayed characteristic resonances for the H-1/C-1 of iridoid [*δ*_H_ 5.20 (1H, d, *J* = 4.5 Hz, H-1), *δ*_C_ 95.3 (C-1)], and the down field of H-3 [*δ*_H_ 7.38 (1H, d, *J* = 1.0 Hz, H-3)] indicated the presence of a 4-substituted enol-ether system. In addition, the 1D NMR spectrum of **1** showed the presence of one methylene signal at [*δ*_H_ 2.72 (1H, dd, *J* = 15.6, 5.5 Hz, H-14a), 2.63 (1H, dd, *J* = 15.6, 6.9 Hz, H-14b); *δ*_C_ 39.3 (C-14)], one oxygenated methine signal at [*δ*_H_ 4.39 (1H, dd, *J* = 12.3, 5.8 Hz, H-15); *δ*_C_ 67.0 (C-15)], one methoxy signal at [*δ*_H_ 3.64 (3H, s, H-17); *δ*_C_ 51.8 (C-17)], and a *β*-d-glucopyranosyl [*δ*_H_ 4.46 (1H, d, *J* = 7.9 Hz, H-1′); *δ*_C_ 98.7 (C-1′), 73.2 (C-2′), 76.8 (C-3′), 70.1 (C-4′), 77.4 (C-5′), 61.2 (C-6′)] (Table 1). The sugar moiety of compound 1 was determined as d-glucose by chiral-HPLC analysis after acid hydrolysis. The above information suggested **1** to be a iridoid glucoside, which was similar to loganin [21], except for the appearance of one methylene [*δ*_H_ 2.72, 2.63 (H-14); *δ*_C_ 39.3 (C-14)], one oxygenated methine [*δ*_H_ 4.39 (H-15); *δ*_C_ 67.0 (C-15)], one methoxy [*δ*_H_ 3.64 (H-17); *δ*_C_ 51.8 (C-17)], and two carbonyl carbons [*δ*_C_ 169.6 (C-13), 173.2 (C-16)]. From the ^1^H-^1^H COSY correlations of H-14 (*δ*_H_ 2.72, 2.63) with H-15 (*δ*_H_ 4.39), it can be deduced that H-14 was directly attached to H-15. In the HMBC spectrum of **1**, correlations of active hydrogen signal (*δ*_H_ 5.80, 15-OH) with C-15 (*δ*_C_ 67.0) and C-16 (*δ*_C_ 173.2) indicated that H-15 was connected to C-16. Moreover, the H-14 (*δ*_H_ 2.72, 2.63) and H-17 (*δ*_H_ 3.64) were determined to be linked to C-13 and C-16 by HMBC correlations of methylene protons at *δ*_H_ 2.72, 2.63 and methoxy protons at *δ*_H_ 3.64 to C-13 (*δ*_C_ 169.6), and C-16 (*δ*_C_ 173.2), respectively. Finally, HMBC correlations from H-7 (*δ*_H_ 5.04) to C-13 (*δ*_C_ 169.6), suggested that the oxygenated methine H-7 was connected to C-13 (Figure 2). In the NOESY spectrum, the correlations between H-1 and H-6 (*δ*_H_ 1.67)/H-8 indicated that H-8 was *α-*oriented, Me-10 was *β*-oriented; the correlations between H-6 (*δ*_H_ 1.67) and H-7 indicated that H-7 was *α-*oriented. Meanwhile, the correlations from H-5 to H-9 and H-6 (*δ*_H_ 2.13) confirmed that H-9 and H-5 were *β-*oriented (Figure 3). Thus, compound **1** was elucidated as shown in Figure 1, and named neocornuside A. More details are shown in Supplementary Materials.

Compound **2** possessed the same molecular formula as **1** according to its HRESIMS at *m/z* 543.1685 [M + Na]^+^ (Calcd. 543.1684). To the structure of compound **2**, except for the chemical shifts of C-14 (**2**, *δ*_C_ 68.8; **1**, *δ*_C_ 39.3) and C-15 (**2**, *δ*_C_ 40.0; **1**, *δ*_C_ 67.0), other signals were very close to those of **1** in the 1D NMR spectra (Table 1). The evident difference was that the methoxy signal H-17 (*δ*_H_ 3.70) was connected to C-15 (*δ*_C_ 40.0) in compound **2** but not C-14 (*δ*_C_ 68.8). The deduction can be further supported by the HMBC correlations between H-17 (*δ*_H_ 3.70) and C-15 (*δ*_C_ 40.0) (Figure 2). The sugar moiety in **2** was also identified as d-glucose by acid hydrolysis and chiral-HPLC analysis. Thus, the structure of compound **2** was defined as shown in Figure 1, and named neocornuside B. More details are shown in Supplementary Materials.

The molecular formula of compound **3**, C_34_H_50_O_20_, was determined based on its HRESIMS at *m/z* 801.2795 [M + Na]^+^ and ^13^C NMR data (Table 2). The NMR data of **3** were consistent with those of cornuside L [21], except for the chemical shifts of C-7 (**3**, *δ*_C_ 79.5; cornuside L,*δ*_C_ 75.1), C-8 (**3**, *δ*_C_ 44.3; cornuside L,*δ*_C_ 42.5), and C-10 (**3**, *δ*_C_ 17.6; cornuside L,*δ*_C_ 13.8). All of the aforementioned information and signals of HMBC, COSY, and HSQC confirm the planar structure of **3** was identical to cornuside L. However, the NOESY correlations (Figure 3) showed that H-5, H-7, H-9, H-10, H-5″, H-7″, H-8″, and H-9″ were *β*-oriented, whereas, H-1, H-8, H-1″, and H-10″ were *α*-oriented. Acid hydrolysis and chiral-HPLC analysis suggested the sugar moiety was d-glucose. Thus, the structure of compound **3** was determined and named neocornuside C, as shown in Figure 1. More details are shown in Supplementary Materials.

The molecular formula of compound **4** was confirmed as C_35_H_52_O_21_ with 10 degrees of unsaturation on the basis of HRESIMS (*m/z*: 831.2913 [M + Na]^+^) and ^13^C NMR (Table 2). Its 1D NMR data (Table 2) were similar to those of cornuside I [21], with the difference of chemical shifts of C-2′ (**4**, *δ*_C_ 73.5; cornuside I,*δ*_C_ 83.1) and C-3′ (**4**, *δ*_C_ 86.8; cornuside I,*δ*_C_ 76.6). The C-3′ of the 7*β*-*O*-methylmorroniside unit was linked to C-7″ of the *α*-morroniside unit by an ether linkage based on the HMBC correlations between H-7″ at *δ*_H_ 4.80 and C-3′ at *δ*_C_ 86.8. The sugar moieties in **4** were also identified as d-glucose by acid hydrolysis and chiral-HPLC analysis. Consequently, the structure of compound **4** was determined and named neocornuside D, as shown in Figure 1.

Along with the above new compounds, six known iridoid glycosides were isolated from fruits of *Cornus officinalis* and identified as 8-epiloganic acid (**5**) [22], 7*α*-*O*-methylmorroniside (**6**) [15], 7-epiloganin (**7**) [23], 7-dehydrologanin (**8**) [24], 7*β*-*O*-methylmorroniside (**9**) [25], loganin (**10**) [21].

### 2.2. Cell Viability of Compounds ***1***–***10*** in Insulin-Induced HepG2 Cells

**Figure 4:**
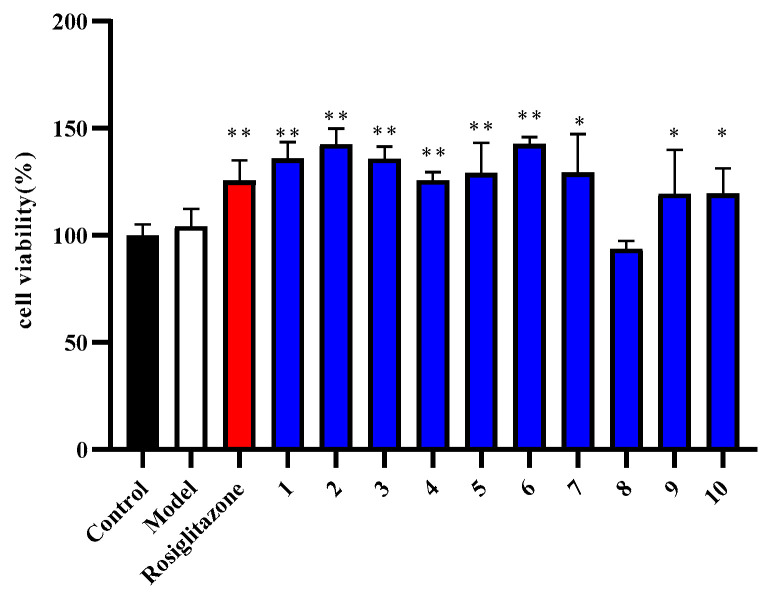
Effect of compounds **1**–**10** on cell viability in insulin-induced HepG2 cells ($\bar{x}$ ± s, n = 4).The impact of compounds **1**–**10** on cell viability measured by CCK-8 assay.* *p* < 0.05 or ** *p* < 0.01, versus insulin group.

Compounds **1**–**10** had no cytotoxic effect on the cell viability of insulin-induced HepG2 cells in the concentration of 10 μM, which was observed by CCK-8 assay (Figure 4). Insulin has mitogenic and anti-apoptotic properties, which promote the progression and metastasis of many types of cancer cells [26,27]. Thus, the compounds and insulin promoted the proliferation of HepG2 cells (Figure 4), and clinical management to counteract insulin resistance and subsequent hyperinsulinemia should be taken to prevent the development of hepatocellular carcinoma (HCC) [28].

### 2.3. Glucose Consumption of Compounds ***1***–***10*** in Insulin-Induced HepG2 Cells 

**Figure 5:**
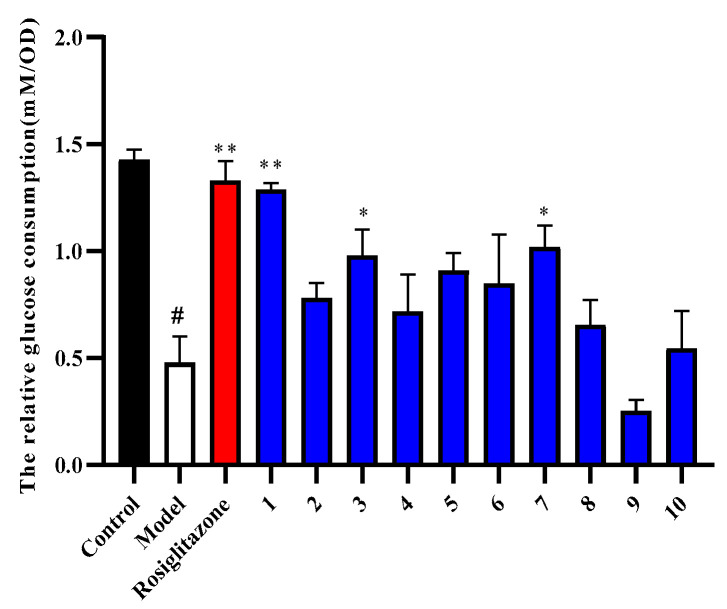
Effect of compounds **1**–**10** on the relative glucose consumption in insulin-induced HepG2 cells ($\bar{x}$ ± s, n = 4). The impact of compounds **1**–**10** on the relative glucose consumption measured by a glucose assay kit. *^#^ p* < 0.05, versus control group; * *p* < 0.05 or ** *p* < 0.01, versus insulin group.

Compounds **1**–**10** were evaluated for their antidiabetic activity against insulin resistant HepG2 cells. The results in Figure 5 showed that compounds **1**, **3**, and **7** significantly increased the relative glucose consumption in insulin-induced HepG2 cells (*p* < 0.05 or *p* < 0.01). Other compounds could increase the relative glucose consumption, but there was no statistical significance compared with the model group (*p* > 0.05). Thus, we focused on the antidiabetic activity of compounds **1**, **3**, and **7**. The results showed that compounds **1**, **3**, and **7** displayed significant antidiabetic activity with EC_50_ values of 0.582, 1.275, and 0.742 µM (Table 3), respectively, which indicate that these effective compounds may improve the insulin resistance in HepG2 cells and could provide reference for the development and application of *C. officinalis* to treat DM. By comparing the structure and activity of compounds **1**, **3**, and **7**, we found that the cyclopentane-type iridoid structural unit may be essential for the antidiabetic activity. Compound **1** showed stronger antidiabetic activity than compounds **3** and **7**, indicating that the activity may be related to the relative configuration at position H-**7**. Moreover, we speculated that compound **3** had an extra *α*-morroniside unit, resulting in lower antidiabetic activity of compound **3** than compound **7**. However, the results showed no significant antidiabetic activity for compounds **2**, **5**, **8**, and **10**, therefore the antidiabetic activity may be related to the relative configuration at position H-**7**, the side chain attached to C-7, and whether the carbonyl group at C-11 forms an ester. In conclusion, further structure-activity relationship remains to be clarified in future research.

### 2.4. Effect of Compounds ***1***, ***3***, and ***7*** on Glucose Uptake in Insulin-Induced HepG2 Cells 

**Figure 6:**
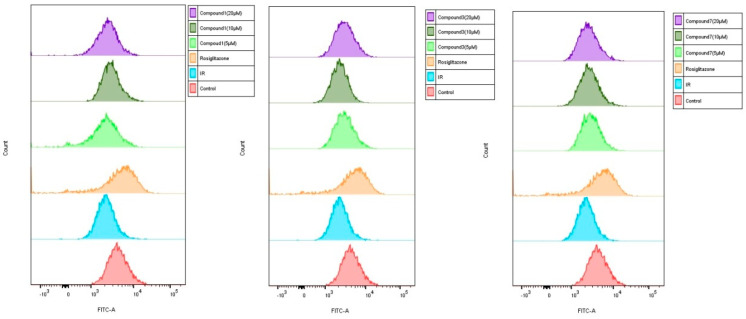
Effect of compounds **1**, **3** and **7** on glucose uptake in insulin-induced HepG2 cells ($\bar{x}$ ± s, n = 4). The 2-NBDG fluorescence measured by flow cytometry.

**Figure 7:**
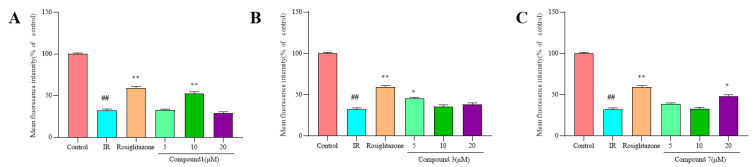
Effect of compounds **1**, **3** and **7** on glucose uptake in insulin-induced HepG2 cells ($\bar{x}$ ± s, n = 4). (**A**) Compound **1** corresponding histograms of the mean fluorescence intensity of 2-NBDG; (**B**) Compound **3** corresponding histograms of the mean fluorescence intensity of 2-NBDG; (**C**) Compound **7** corresponding histograms of the mean fluorescence intensity of 2-NBDG. *^##^ p* < 0.01, versus control group; * *p* < 0.05 or ** *p* < 0.01, versus insulin group.

To investigate whether iridoid glycosides could promote glucose uptake in HepG2 cells, the uptake of 2-NBDG was evaluated by HepG2 cells treated with different concentrations (5, 10 and 20 µM) of test compounds **1**, **3**, and **7**. The results showed that 2-NBDG uptake in HepG2 cells was significantly decreased after exposed to insulin (Figure 6 and Figure 7). However, compounds **1**, **3**, and **7** improved the ability of 2-NBDG uptake in insulin-induced HepG2 cells (Figure 6 and Figure 7). Therefore, compounds **1**, **3**, and **7** efficiently alleviated the HepG2 cells injury induced by insulin, which present potential anti-diabetic effects.

## 3. Experimental 

### 3.1. General Experimental Procedures

CD spectra were recorded on an Applied PhotophysicsChirascanqCD spectropolarimeter (Applied Photophysics Ltd., British). Optical rotations were recorded by using a Rudolph AP-IV polarimeter (Rudolph, Hackettstown, NJ, USA). NMR spectra were recorded by a Bruker Avance III 500 spectrometer (Bruker, Berlin, Germany). UV spectra were measured with a ThermoEVO 300 spectrometer (Thermo, Waltham, MA, USA). IR spectra were detected using a Thermo Nicolet IS 10 spectrometer (Thermo, Waltham, MA, USA). HRESIMS spectra were measured on a Bruker maxis HD mass spectrometer (Bruker, Germany). Sephadex LH-20 (GE Healthcare, Boston, MA, USA), Toyopearl HW-40C (TOSOH, Tokyo, Japan), C_18_ ODS (50 μm, YMC Co., Ltd., Kyoto, Japan), MCI gel CHP-20, macroporous resin Diaion HP-20 (Mitsubishi Chemical Corporation, Tokyo, Japan), and silica gel (100~200 mesh, 200~300 mesh, Qingdao Marine Chemical Co., Ltd., Qingdao, China) were employed for column chromatography (CC). The samples were prepared by a Saipuruisi LC 52 HPLC system with a UV/vis 50 detector (Saipuruisi, Beijing, China) and a YMC-Pack ODS-A column (20 × 250 mm, 5 µm; YMC, Kyoto, Japan). The chemical reagents were supplied by the Tianjin Fuyu (Tianjin, China) and the Tianjin NO. 3 Reagent Plant (Tianjin, China).

### 3.2. Plant Materials

Fruits of *Cornus officinalis* were collected in Xixia county, Henan Province, China, in September 2019, and identified by Prof. Chengming Dong, School of Pharmacy, Henan University of Chinese Medicine. The voucher specimen (NO. 20190903) was preserved in the Department of Pharmaceutical Chemistry, Henan University of Chinese Medicine.

### 3.3. Extraction and Isolation

Fruit flesh of *Cornus officinalis* (100.0 kg) were extracted twice with a 10-fold amount of deionized water under reflux for 2 h each time. The crude extract (55.1 kg) was dissolved in water and subjected to a D101 macroporous adsorption resin column eluting with water and 95% ethanol. The 95% ethanol extract (21.7 kg) was dissolved in water and partitioned with petroleum ether, EtOAc, and *n*-BuOH, successively. The EtOAc extract (99.3 g) was subjected to a Diaion HP-20 macroporous adsorption resin column (90 × 9 cm, 4 × 5700 mL) and eluted with a gradient of EtOH/water (0:100, 20:80, 40:60, 60:40, 95:5, 100:0, *v/v*) to obtain five fractions (F1–F5). F3 (19.6 g) was chromatographed over a Sephadex LH-20 column eluting with gradients of MeOH/H_2_O (20:80, 50:50, 100:0, *v/v*) to afford five fractions (F3.1–F3.5). F3.1 (16.0 g) was loaded on a silica gel column eluting with petroleum ether/acetone (3:1, 2:1, 0:1, *v/v*) to yield six fractions (F3.1.1–F3.1.6). F3.1.5 (650.0 mg) was further purified on the semi-preparative HPLC with MeOH/water (35:65, *v/v*; 3 mL/min) to yield compound **6** (145.8 mg, *t*_R_ = 41.1 min). F3.1.4 (3.5 g) was applied to a silica gel column and eluted with a gradient of CH_2_Cl_2_/MeOH (15:1, 10:1, 5:1, *v/v*) to obtain six fractions (F3.1.4.1–F3.1.4.6). F3.1.4.1 (450.5 mg) was purified by the semi-preparative HPLC with MeCN/water (17:83, *v/v*; 3 mL/min) to obtain compound **10** (50.6 mg, *t*_R_ = 9.2 min). By employing the semi-preparative HPLC with MeCN/water (13:87, *v/v*; 3 mL/min), compounds **8** (17.7 mg, *t*_R_ = 16.9 min) and **9** (315.3 mg, *t*_R_ = 45.8 min) from F3.1.4.2 (652.5 mg) were obtained.

The *n*-BuOH extract (4.2 kg) was applied to Diaion HP-20 macroporous adsorption resin and eluted with a gradient of EtOH/water (0:100, 20:80, 40:60, 95:5, 100:0, *v/v*) to obtain four fractions (A1–A4). A2 (201.0 g) was subjected to MCI column eluting with MeOH/water (0:100, 20:80, 40:60, 60:40, 100:0, *v/v*) to give five fractions (A2.1–A2.5). A2.1 (65.6 g) was then split by MCI column eluting with MeOH/water (0:100, 20:80, 30:70, 40:60, 50:50, 70:30, 100:0, *v/v*) to afford five fractions (A2.1.1–A2.1.5). A2.1.3 (15.7 g) was subjected to a silica gel (200–300 mesh) column eluting with CH_2_Cl_2_/MeOH (12:1, 10:1, 8:1, *v/v*) to produce five fractions (A2.1.3.1–A2.1.3.5). A2.1.3.1.4 (1.4 g) was separated and purified on a silica gel column eluting with CH_2_Cl_2_/MeOH (15:1, 10:1, 5:1, 1:1, *v/v*) and a further purification by the semi-preparative HPLC with MeCN/H_2_O (6:94, *v/v*; 3 mL/min) to give compounds **5** (25.6 mg, *t*_R_ = 47.7 min) and **7** (3.6 mg, *t*_R_ = 32.6 min). A2.1.4 (44.3 g) was split on a Sephadex LH-20 column eluting with MeOH/H_2_O (40:60, 60:40, 80:20, *v/v*) to obtain nine fractions (A2.1.4.1–A2.1.4.9). A2.1.4.2 (5.3 g) was applied to MCI column and eluted with a gradient of MeOH/H_2_O (20:80, 40:60, 60:40, 80:20, 100:0, *v/v*) to give seven fractions (A2.1.4.2.1–A2.1.4.2.7). Compound **1** (44.1 mg, *t*_R_ = 49.3 min) was obtained from A2.1.4.2.4 (890.0 mg) by a silica gel (200–300 mesh) column eluted with CH_2_Cl_2_/MeOH (20:1, 15:1, 10:1, *v/v*) followed by a final purification on the semi-preparative HPLC (MeCN/H_2_O, 14:86, *v/v*; 3 mL/min). A2.1.4.3 (1.0 g) was subjected to Sephadex LH-20 column eluting with MeOH to give two fractions (A2.1.4.3.1–A2.1.4.3.2). A2.1.4.3.1 (350.5 mg) was purified by the semi-preparative HPLC with MeCN/H_2_O (16:84, *v/v*; 3 mL/min) to obtain compound **2** (7.0 mg, *t*_R_ = 27.1 min). A2.1.4.2.6 (1.5 g) was applied to a silica gel column eluting with CH_2_Cl_2_/MeOH (20:1, 15:1, 10:1, 5:1, 1:1, *v/v*) to yield four fractions (A2.1.4.2.6.1–A2.1.4.2.6.4). A2.1.4.2.6.2 (300.5 mg) was further purified on the semi-preparative HPLC with MeCN/H_2_O (20:80, *v/v*; 3 mL/min) to give compound **4** (3.6 mg, *t*_R_ = 47.9min). A2.2 (74.0 g) was subjected to Toyopearl HW-40C column eluting with MeOH to produce three fractions (A2.2.1–A2.2.3). A2.2.1 (20.5 g) was chromatographed over a Toyopearl HW-40Ccolumn eluting with gradients of MeOH/H_2_O (20:80, 50:50, 70:30, 100:0, *v/v*) to afford seven fractions (A2.2.1.1–A2.2.1.7). A2.2.1.1 (2.6 g) was applied to a silica gel column eluting with CH_2_Cl_2_/MeOH (12:1, 8:1, *v/v*) to get five fractions (A2.2.1.1.1–A2.2.1.1.5), then A2.2.1.1.3 (610.5 mg) was purified by the semi-preparative HPLC with MeOH/H_2_O (34:66, *v/v*; 3 mL/min) to give compound **3** (2.7 mg, *t*_R_ = 44.8 min).

Neocornuside A (**1**): White amorphous power (MeOD); HRESIMS *m/z*: 543.1689 [M + Na]^+^ (Calcd. for C_22_H_32_O_14_Na, 543.1684); [*α*]_D_^20^ −36 (*c* 1.25, MeOH); CD (MeOH) Δε: 227 (−1.09) nm; UV (MeOH) *λ*max (log *ε*): 200 (4.05), 228 (3.51) nm; IR *ν*_max_: 3393, 2952, 1731 cm^−1^; ^1^H NMR (500 MHz, DMSO-*d*_6_) and ^13^C NMR (125 MHz, DMSO-*d*_6_), see Table 1.

Neocornuside B (**2**): White amorphous power (MeOD); HRESIMS *m/z*: 543.1685 [M + Na]^+^ (Calcd. for C_22_H_32_O_14_Na, 543.1684); [*α*]_D_^20^ −45 (*c* 0.16, MeOH); CD (MeOH) Δε: 227 (−2.85) nm; UV (MeOH) *λ*max (log *ε*): 234 (3.86) nm; IR *ν*_max_: 3379, 2927, 1697 cm^−1^; ^1^H NMR (500 MHz, MeOD) and ^13^C NMR (125 MHz, MeOD), see Table 1.

Neocornuside C (**3**): White amorphous power (MeOD); HRESIMS *m/z*: 801.2795 [M + Na]^+^ (Calcd. for C_34_H_50_O_20_Na, 801.2788); [α]_D_^20^ −68 (*c* 0.13, MeOH); CD (MeOH) Δε: 229 (−2.21) nm; UV (MeOH) *λ*max (log *ε*): 196 (4.12), 238 (4.13) nm; IR *ν*_max_: 3368, 2932, 1694, 1638 cm^−1^; ^1^H NMR (500 MHz, MeOD) and ^13^C NMR (125 MHz, MeOD), see Table 2.

Neocornuside D (**4**): White amorphous power (MeOH); HRESIMS *m/z*: 831.2912 [M + Na]^+^ (Calcd. for C_35_H_52_O_21_Na, 831.2893); [α]_D_^20^ −94 (*c* 0.07, MeOH); CD (MeOH) Δε: 233 (−1.97) nm; UV (MeOH) *λ*max (log *ε*): 194 (4.22), 236 (4.13) nm; IR *ν*_max_: 3383, 2923, 1690, 1638 cm^−1^; ^1^H NMR (500 MHz, MeOD) and ^13^C NMR (125 MHz, MeOD), see Table 2. 

### 3.4. Antidiabetic Evaluation

#### 3.4.1. Cell Culture and Treatment

HepG2 (human hepatocellular liver carcinoma) cells were a gift from QiujunLv of the Radiation Academy of Military Medical Sciences (Beijing, China). The HepG2 cells were cultured at 37 °C in high glucose DMEM medium containing with 15% FSP500 fetal bovine serum in a humidified atmosphere of 5% CO_2_. The cells were seeded in 96-well plates at a density of 1 × 10^5^ cells/well. To induce insulin resistance (IR), HepG2 cells were starved in serum-free DMEM for 24 h. One day after cultivating, the medium was changed to serum-free DMEM containing 20 μM insulin and incubated for a further 24 h. Afterwards, the DMEM medium, the medium with rosiglitazone (10 µM), or the medium with test compounds (**1**–**10**) (10 µM) were added, respectively, followed by the incubation for 24 h. In addition, HepG2 cells were also treated with test compounds **1**, **3**, **7**, and rosiglitazone in different concentrations (0.625, 1.25, 2.5, 5, 10, 20, and 30 µM), then incubated at 37 °C for 24 h. The EC_50_ values were calculated by the software of Graphpad Prim 8.

#### 3.4.2. Cell Viability Assay

CCK-8 assay was used to evaluate cell viability of HepG2 cells. After treatments, added 10 μL of CCK-8 reagent and incubated at 37 °C for 1 h. The optical density (OD) value of every well was measured at 450 nm using a microplate spectrophotometer. 

#### 3.4.3. Glucose Consumption Assay

The HepG2 cells (1 × 10^5^ cells/mL) cultured in 96-well plates were treated with insulin and test compounds as previously described. The culture medium was collected, and the glucose concentrations were measured using the same method [29] with a glucose assay kit. The glucose content of the experimental group medium was subtracted from the glucose content of the original DMEM medium to afford the glucose consumption (GC). The relative glucose consumption (RGC) was calculated by the following formula: RGC = GC/OD.

#### 3.4.4. Glucose Uptake Assay

Glucose uptake rate was measured using 2-NBDG, according to the previously reported method [29]. The HepG2 cells were seeded at 1 × 10^5^ cells/mL in 6-well plates at 37 °C for 24 h in a humidified atmosphere of 5% CO_2_. The cells were pre-incubated with various concentrations of test compounds **1**, **3**, and **7** (5, 10, and 20 µM). After 24 h, 2-NBDG (25 μM) was added in incubation at 37 °C for 1 h. The cells were collected and washed with PBS, and then resuspended in PBS. The cell fluorescence intensity was detected by flow cytometry with excitation wavelength of 488 nm and emission wavelength of 530 nm. The results were analyzed by the software FlowJo 10.8.

### 3.5. Acid Hydrolysis of Compounds ***1***–***4***

Each one (1.0 mg) of the compounds **1**–**4** was dissolved in 2 M HCl*-*H_2_O (2.5 mL) and heated at 80 °C for 3 h. The reaction mixture was extracted with EtOAc. The aqueous layer was evaporated under vacuum, diluted repeatedly with H_2_O, and evaporated in vacuo to furnish a neutral residue. The residue was dissolved in MeOH (1.5 mL) and analyzed by HPLC equipped with a chiral column (CHIRALPAK AD-H, 5 μm, 4.6 × 250 mm) and an evaporative light scattering detector using n-hexane-EtOH (82:18; *v/v*) as the mobile phase (0.5 mL/min). For all of the selected compounds, the sugars were found to be d-glucoses by comparing its retention time with that of d-glucose (21.413 and 22.554 min) and l-glucose (22.099 and 23.648 min).

## 4. Conclusions

In summary, the chemical composition of *Cornus officinalis*fruit was further investigated, leading to the isolation of four new iridoid glycosides, neocornuside A–D (**1**–**4**), together with six known compounds (**5**–**10**). Among the isolated compounds, no cytotoxic effect was seen on the cell viability of insulin-induced HepG2 cells in the concentration of 10 µM, and compounds **1**, **3**, and **7** displayed significant antidiabetic activity with EC_50_ values of 0.582, 1.275, and 0.742 µM, respectively, which was proven to have the potential to ameliorate the glucose uptake of insulin-induced HepG2 cells in doses of 10, 5, and 20 µM, respectively. These effective compounds may represent promising natural antidiabetic compounds for the treatment of DM. It also provided scientific evidence and a foundation for the understanding of the antidiabetic effects and further utilization of *Cornus officinalis*. In future research, enrichment of the active compounds should be performed for in vivo validation. On the other hand, other compounds will be investigated further for their potential activity using a Surface Plasmon Resonance (SPR) technique for expanding the scope of application.

## Figures and Tables

**Table 1:** ^1^H NMR (500 MHz) and ^13^C NMR (125 MHz) data (*δ* in ppm, *J* in Hz) for compounds **1**–**2.**

	1 *^a^*		2 *^b^*	
Position	^13^C	^1^H (*J* in Hz)	^13^C	^1^H (*J* in Hz)
1	95.3	5.20 d (4.5)	97.5	5.29 d (4.9)
3	150.9	7.38 d (1.0)	152.6	7.43d (1.3)
4	111.4		113.1	
5	30.7	2.94 m	32.6	3.10 q (8.0)
6	38.7	2.13 m 1.67 m	40.3	2.37 ddd (14.7, 8.0, 1.5) 1.76 ddd (14.7, 8.0, 5.0)
7	77.0	5.04 m	79.9	5.21 t (4.6)
8	38.9	1.97 m	40.9	2.14 m
9	45.2	1.92 m	46.9	2.07 td (8.9, 4.9)
10	13.1	0.95 d (6.7)	13.6	1.07 d (6.8)
11	166.8		169.3	
12	51.1	3.62 s	52.3	3.69 s
13	169.6		174.1	
14	39.3	2.72 dd (15.6, 5.5) 2.63 dd (15.6, 6.9)	68.8	4.52 dd (7.0, 5.2)
15	67.0	4.39, dd (12.3, 5.8)	40.0	2.83 dd (16.0, 5.2) 2.74 dd (16.0, 7.0)
16	173.2		172.4	
17	51.8	3.64 s	51.7	3.70 s
1′	98.7	4.46 d (7.9)	100.2	4.66 d (7.9)
2′	73.2	2.97 m	74.7	3.19 m
3′	76.8	3.15 m	78.0	3.37 t (8.9)
4′	70.1	3.03 m	71.6	3.26 m
5′	77.3	3.14 m	78.4	3.32 m
6′	61.2	3.68 m 3.44 m	62.8	3.90 dd (11.9, 2.1) 3.65 dd (11.9, 6.2)

*^a^* In DMSO-*d*_6_; *^b^* In MeOD.

**Table 2:** ^1^H NMR (500 MHz) and ^13^C NMR (125 MHz) data (*δ* in ppm, *J* in Hz) for compounds **3**–**4** *^a^*.

	3		4	
Position	^13^C	^1^H (*J* in Hz)	^13^C	^1^H (*J* in Hz)
1	97.9	5.24 d (5.3)	95.4	5.91 d (3.2)
3	152.4	7.41 s	154.5	7.53 s
4	113.3		111.7	
5	31.2	2.88 m	28.0	3.10 dt (12.9, 4.7)
6	41.9	2.51 dt (12.9, 7.4) 1.34 m	33.8	1.93 dd (13.9, 4.7) 1.52 td (13.9, 3.9)
7	79.5	3.68 m	99.5	4.74 d (3.7)
8	44.3	1.72 q (7.5)	66.3	4.29 qd (6.6, 2.1)
9	46.9	1.84 m	40.4	1.83 m
10	17.6	1.16 d (6.7)	19.6	1.34 d (6.9)
11	169.5		168.7	
12	51.8	3.69 s	51.8	3.70 s
13			55.0	3.35 s
1′	100.6	4.65 d (8.0)	99.7	4.87 d (8.7)
2′	74.7	3.22 m	73.5	3.25 m
3′	78.5	3.29 m	86.8	3.57 t (8.9)
4′	71.0	3.40 m	70.4	3.38 m
5′	78.0	3.38 m	78.0	3.37 m
6′	68.7	4.02 m 3.93 dd (11.9, 1.5)	62.6	3.90 dd (12.2, 1.8) 3.87 dd (12.2, 6.5)
1″	95.9	5.82 d (9.2)	96.1	5.89 d (3.3)
3″	154.5	7.52 s	154.6	7.52 s
4″	110.8		110.8	
5″	31.9	2.85 m	32.1	2.87 dt (12.9, 4.6)
6″	35.7	2.07 ddd (13.2, 4.6, 2.2) 3.65 td (13.2, 9.7)	35.4	2.26 ddd (13.4, 4.6, 2.4) 1.32 m
7″	104.0	4.71 dd (9.7, 2.2)	103.8	4.80 dd (9.7, 2.4)
8″	74.3	3.98 dd (6.9, 2.3)	74.5	4.05 qd (6.4, 1.7)
9″	40.1	1.81 m	40.0	1.81 m
10″	19.7	1.41 d (6.80)	19.7	1.45 d (6.8)
11″	168.6		168.6	
12″	51.7	3.70 s	51.7	3.71 s
1‴	100.0	4.78 d (7.8)	100.4	4.79 d (7.9)
2‴	75.1	3.23 m	75.1	3.22 m
3‴	77.7	3.39 m	78.6	3.28 d (2.3)
4‴	71.7	3.27 m	71.6	3.27 m
5‴	77.0	3.41 m	78.4	3.33 m
6‴	62.9	3.89 dd (12.2, 2.2) 3.66 m	62.7	3.66 m

*^a^* In MeOD.

**Table 3:** Effect of compounds **1**, **3**, and **7** on the relative glucose consumption in insulin-induced HepG2 cells ($\bar{x}$ ± s, n = 6).

Compound	EC_50_ *^a^* (μM)
**1**	0.582
**3**	1.275
**7**	0.742
Rosiglitazone *^b^*	1.127

*^a^*EC_50_value of each compound was defined as the concentration (μM) for 50% maximal effect of the relative glucose consumption in insulin-induced HepG2 cells. *^b^*Rosiglitazone: positive control.

## Data Availability

The data presented in this study are available in the Supplementary Materials.

## Supplementary Materials

The following supporting information can be downloaded at: https://www.mdpi.com/article/10.3390/molecules27154732/s1. Scheme S1: Extraction and isolation flow chart of compounds **1**–**10** from fruits of *Cornus officinalis*. Figures S1–S7: ^1^H NMR, ^13^C NMR and 2D NMR spectra of compound **1**, Figures S8–S11: UV, IR, CD and HRESIMS spectra of compound **1**; Figures S12–S18: ^1^H NMR, ^13^C NMR and 2D NMR spectra of compound **2**; Figures S19–S22: UV, IR, CD and HRESIMS spectra of compound **2**; Figures S23–S29: ^1^H NMR, ^13^C NMR and 2D NMR spectra of compound **3**; Figures S30–S33: UV, IR, CD and HRESIMS spectra of compound **3**; Figures S34–S40: ^1^H NMR, ^13^C NMR and 2D NMR spectra of compound **4**; Figures S41–S44: UV, IR, CD and HRESIMS spectra of compound **4**; Figures S45–S56: ^1^H NMR, ^13^C NMR spectra of compounds **5**–**10**; Tables S1–S2: The results of antidiabetic evaluation.

## Sample Availability

Samples of the compounds are available from the authors.
