# Supplementary material for: Neocornuside A–D, Four Novel Iridoid Glycosides from Fruits of Cornus officinalis and Their Antidiabetic Activity
Source: Molecules. 2022 Jul 24;27(15):4732. doi: 10.3390/molecules27154732 (PMC9331380; doi:10.3390/molecules27154732)
Supplement: Supplementary file 1 [file molecules-27-04732-s001.zip › molecules-1813861-supplementary.pdf]

Neocornuside A–D, Four Novel Iridoid Glycosides from Fruits of *Cornus officinalis* and Their Antidiabetic Activity

Meng Yang [1,3], Zhiyou Hao [1,3], Xiaolan Wang [2,3], Shiqi Zhou [1,3], Denghui Zhu [1,3], Ying Yang [1,3], Junjun Wei [1,3], Meng Li [1,3], Xiaoke Zheng [1,3*] and Weisheng Feng [1,3*]

[1] *School of Pharmacy, Henan University of Chinese Medicine, Zhengzhou 450046, China*

[2] *Academy of Chinese Medical Sciences, Henan University of Chinese Medicine, Zhengzhou 450046, China*

[3] *The Engineering and Technology Center for Chinese Medicine Development of Henan Province China, Zhengzhou 450046, China*

[*] Corresponding authors: Xiaoke Zheng (X.K.Z.); Weisheng Feng (W.S.F.)
E-mail addresses: zhengxk.2006@163.com (X.K.Z.); fwsh@hactcm.edu.cn (W.S.F.)

Contents

Scheme S1. Extraction and isolation flow chart of compounds **1–10** from fruits of *Cornus officinalis*....4
Figure S1. ^{1}H NMR spectrum of compound **1** (500 MHz, DMSO-d_6)....5
Figure S2. ^{13}C NMR spectrum of compound **1** (125 MHz, DMSO-d_6)....5
Figure S3. HSQC spectrum of compound **1**....6
Figure S4. DEPT135 spectrum of compound **1**....6
Figure S5. ^{1}H-^{1}H COSY spectrum of compound **1**....7
Figure S6. HMBC spectrum of compound **1**....7
Figure S7. NOESY spectrum of compound **1**....8
Figure S8. UV spectrum of compound **1**....9
Figure S9. IR spectrum of compound **1**....9
Figure S10. CD spectrum of compound **1**....9
Figure S11. HRESIMS spectrum of compound **1**....10
Figure S12. ^{1}H NMR spectrum of compound**2** (500 MHz, MeOD)....11
Figure S13. ^{13}C NMR spectrum of compound **2** (125 MHz, MeOD)....11
Figure S14. HSQC spectrum of compound **2**....12
Figure S15. DEPT135 spectrum of compound **2**....12
Figure S16. ^{1}H-^{1}H COSY spectrum of compound **2**....13
Figure S17. HMBC spectrum of compound **2**....13
Figure S18. NOESY spectrum of compound **2**....14
Figure S19. UV spectrum of compound **2**....15
Figure S20. IR spectrum of compound **2**....15
Figure S21. CD spectrum of compound **2**....16
Figure S22. HRESIMS spectrum of compound **2**....17
Figure S23. ^{1}H NMR spectrum of compound **3** (500 MHz, MeOD)....18
Figure S24. ^{13}C NMR spectrum of compound **3** (125 MHz, MeOD)....18
Figure S25. HSQC spectrum of compound **3**....19
Figure S26. DEPT135 spectrum of compound **3**....19
Figure S27. ^{1}H-^{1}H COSY spectrum of compound **3**....20
Figure S28. HMBC spectrum of compound **3**....20
Figure S29. NOESY spectrum of compound **3**....21
Figure S30. UV spectrum of compound **3**....22
Figure S31. IR spectrum of compound **3**....22
Figure S32. CD spectrum of compound **3**....23
Figure S33. HRESIMS spectrum of compound **3**....24
Figure S34. ^{1}H NMR spectrum of compound **4** (500 MHz, MeOD)....25
Figure S35. ^{13}C NMR spectrum of compound **4** (125 MHz, MeOD)....25
Figure S36. HSQC spectrum of compound **4**....26
Figure S37. DEPT135 spectrum of compound **4**....26
Figure S38. ^{1}H-^{1}H COSY spectrum of compound **4**....27
Figure S39. HMBC spectrum of compound **4**....27
Figure S40. NOESY spectrum of compound **4**....28

Figure S41. UV spectrum of compound **4** 28
Figure S42. IR spectrum of compound **4** 29
Figure S43. CD spectrum of compound **4** 29
Figure S44. HRESIMS spectrum of compound **4** 30
Figure S45. ^{1}H NMR spectrum of compound **5** (500 MHz, MeOD) 31
Figure S46. ^{13}C NMR spectrum of compound **5** (125 MHz, MeOD) 31
Figure S47. ^{1}H NMR spectrum of compound **6** (500 MHz, MeOD) 32
Figure S48. ^{13}C NMR spectrum of compound **6** (125 MHz, MeOD) 32
Figure S49. ^{1}H NMR spectrum of compound **7** (500 MHz, MeOD) 33
Figure S50. ^{13}C NMR spectrum of compound **7** (125 MHz, MeOD) 33
Figure S51. ^{1}H NMR spectrum of compound **8** (500 MHz, MeOD) 34
Figure S52. ^{13}C NMR spectrum of compound **8** (125 MHz, MeOD) 34
Figure S53. ^{1}H NMR spectrum of compound **9** (500 MHz, MeOD) 35
Figure S54. ^{13}C NMR spectrum of compound **9** (125 MHz, MeOD) 35
Figure S55. ^{1}H NMR spectrum of compound **10** (500 MHz, MeOD) 36
Figure S56. ^{13}C NMR spectrum of compound **10** (125 MHz, MeOD) 36
Table S1. Effect of compounds **1–10** on the relative glucose consumption in insulin-induced HepG2 cells (x ± s, n = 4). 37
Table S2. Effect of compounds **1**, **3**, and **7** on the relative glucose consumption in insulin-induced HepG2 cells (x ± s, n = 6). 38

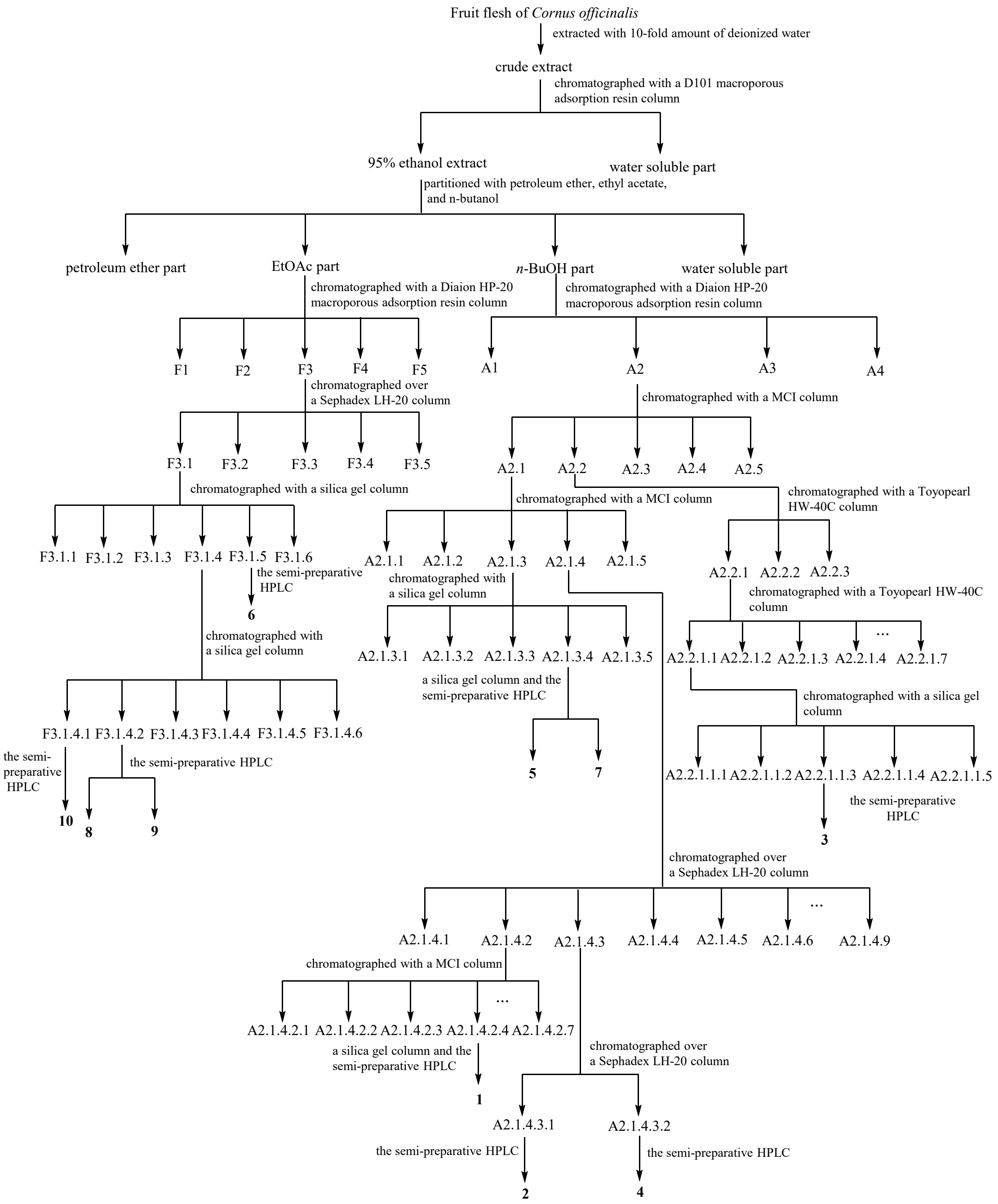

Scheme S1. Extraction and isolation flow chart of compounds **1**–**10** from fruits of *Cornus officinalis*

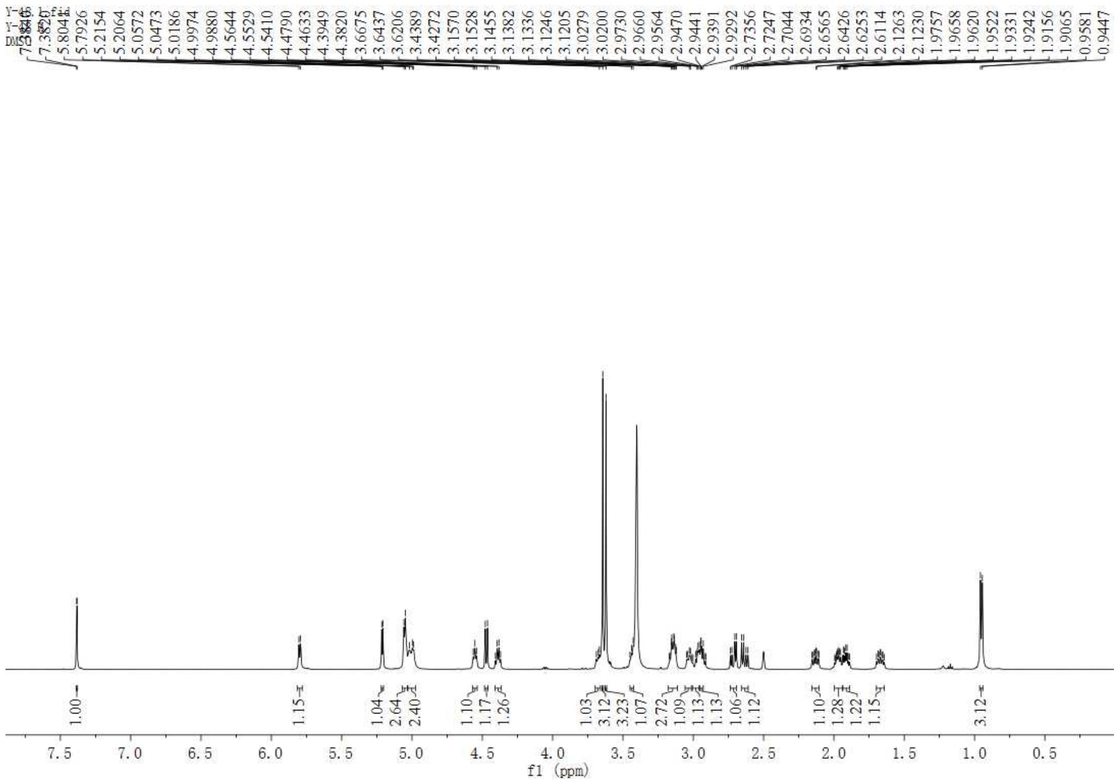

Figure S1. ^{1}H NMR spectrum of compound **1** (500 MHz, DMSO-d_6)

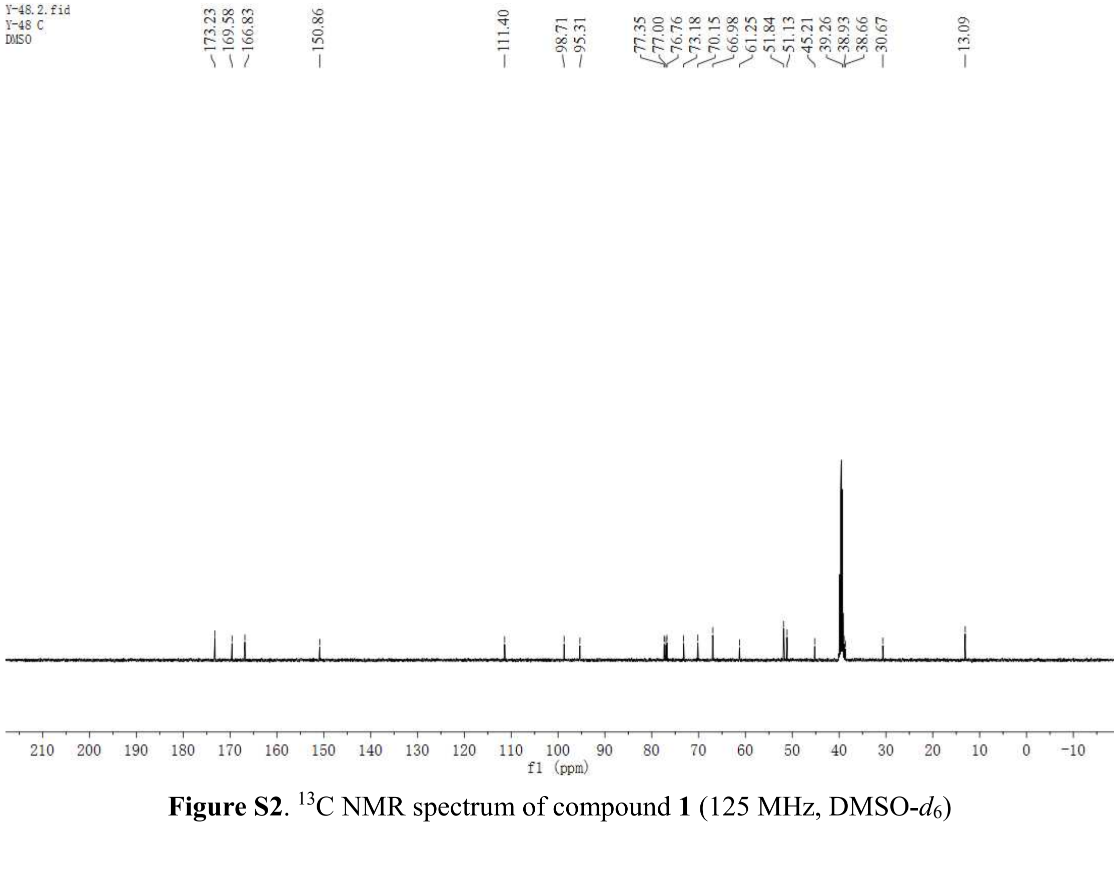

Figure S2. ^{13}C NMR spectrum of compound **1** (125 MHz, DMSO-d_6)

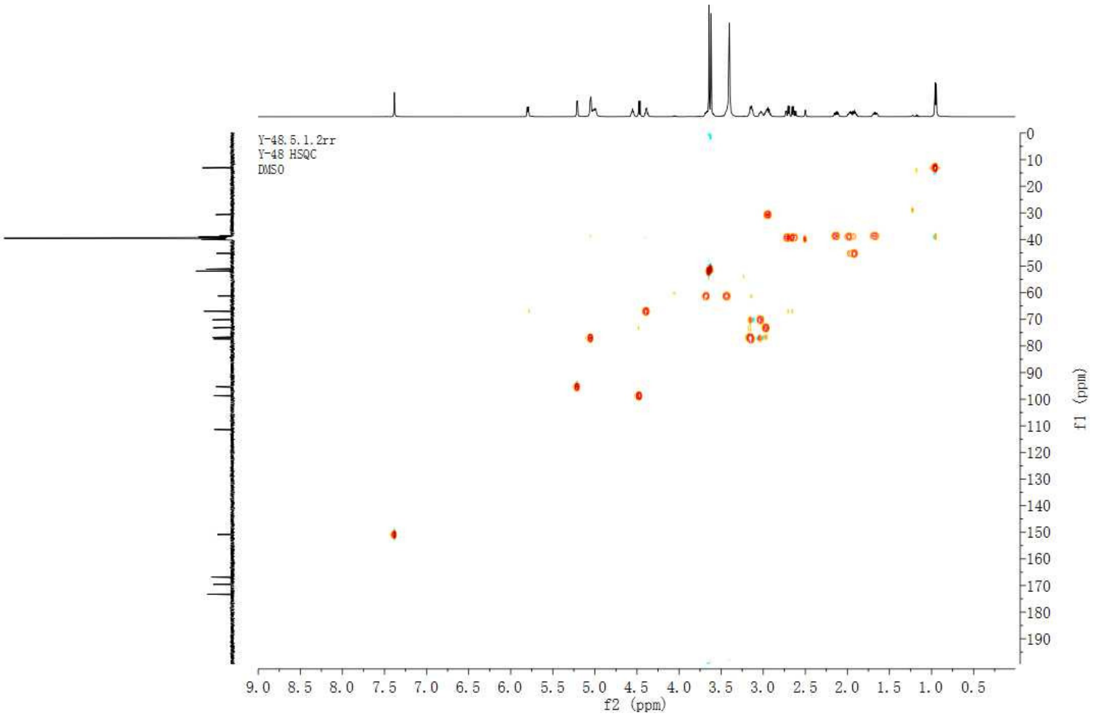

Figure S3. HSQC spectrum of compound **1**

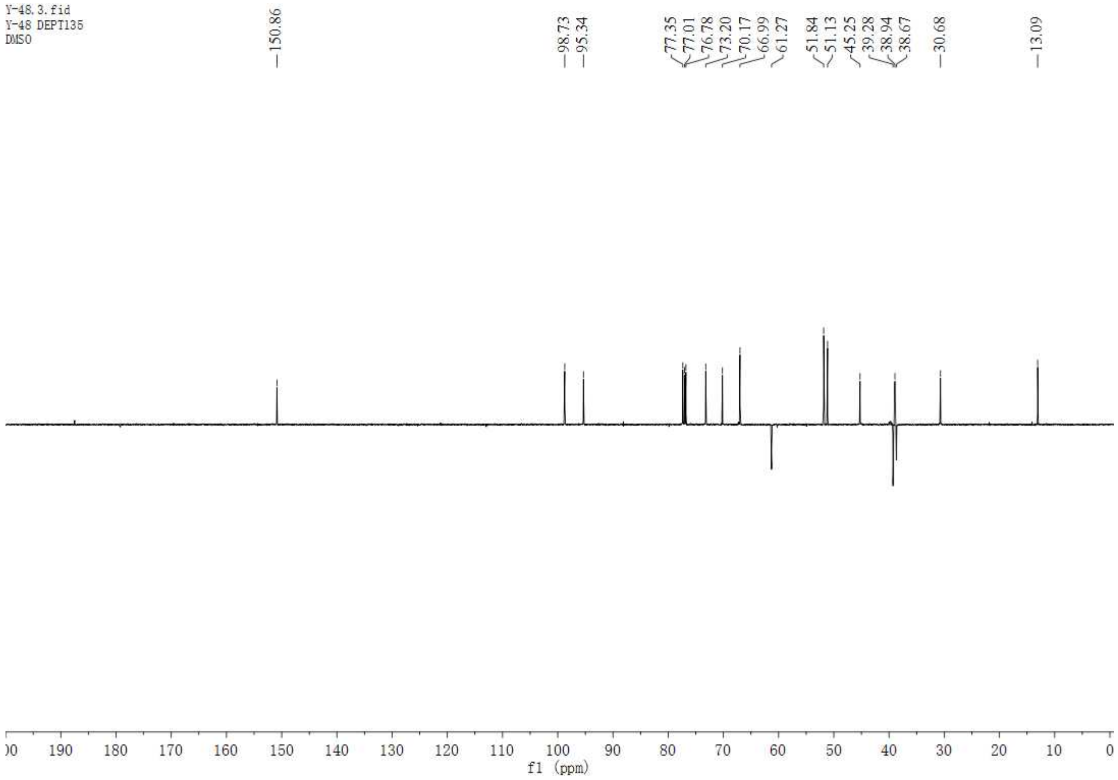

Figure S4. DEPT135 spectrum of compound **1**

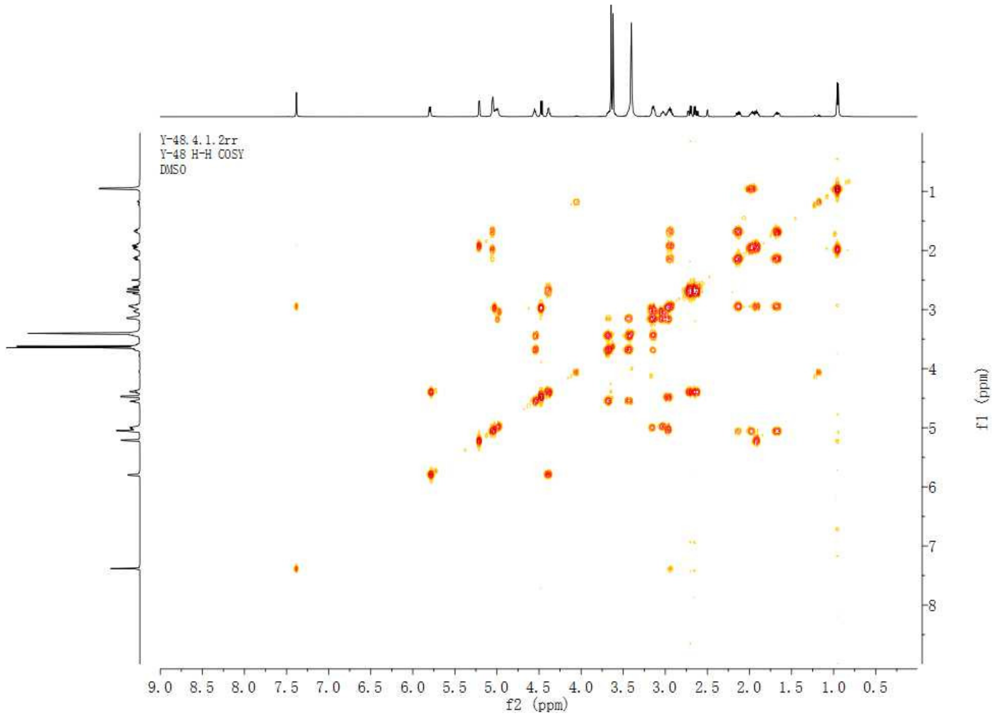

Figure S5. ^{1}H-^{1}H COSY spectrum of compound **1**

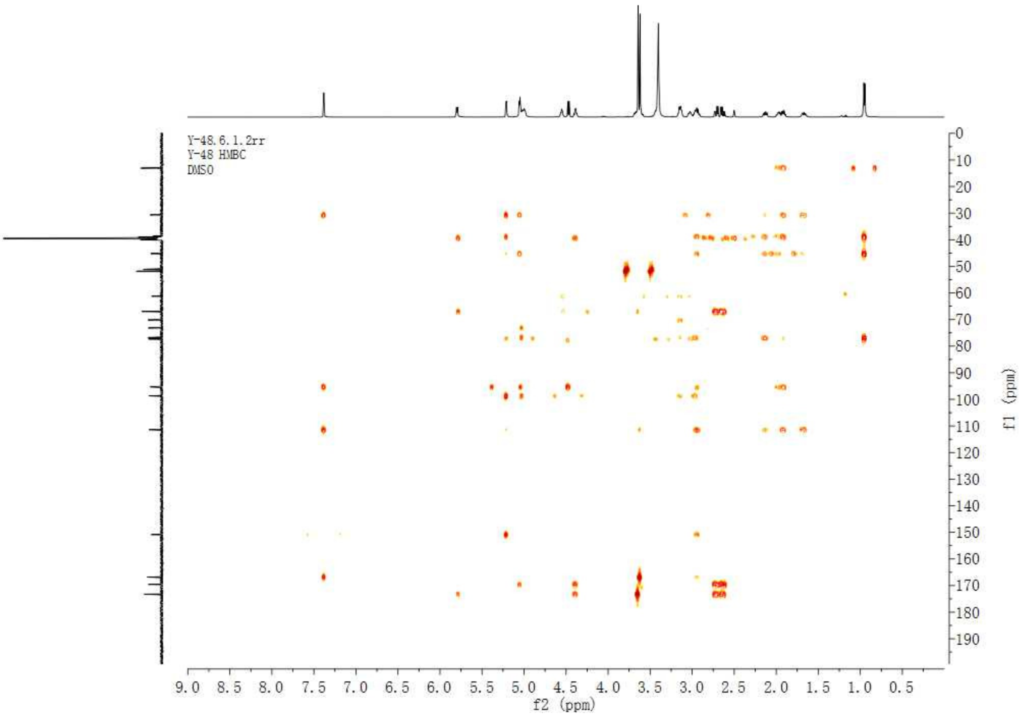

Figure S6. HMBC spectrum of compound **1**

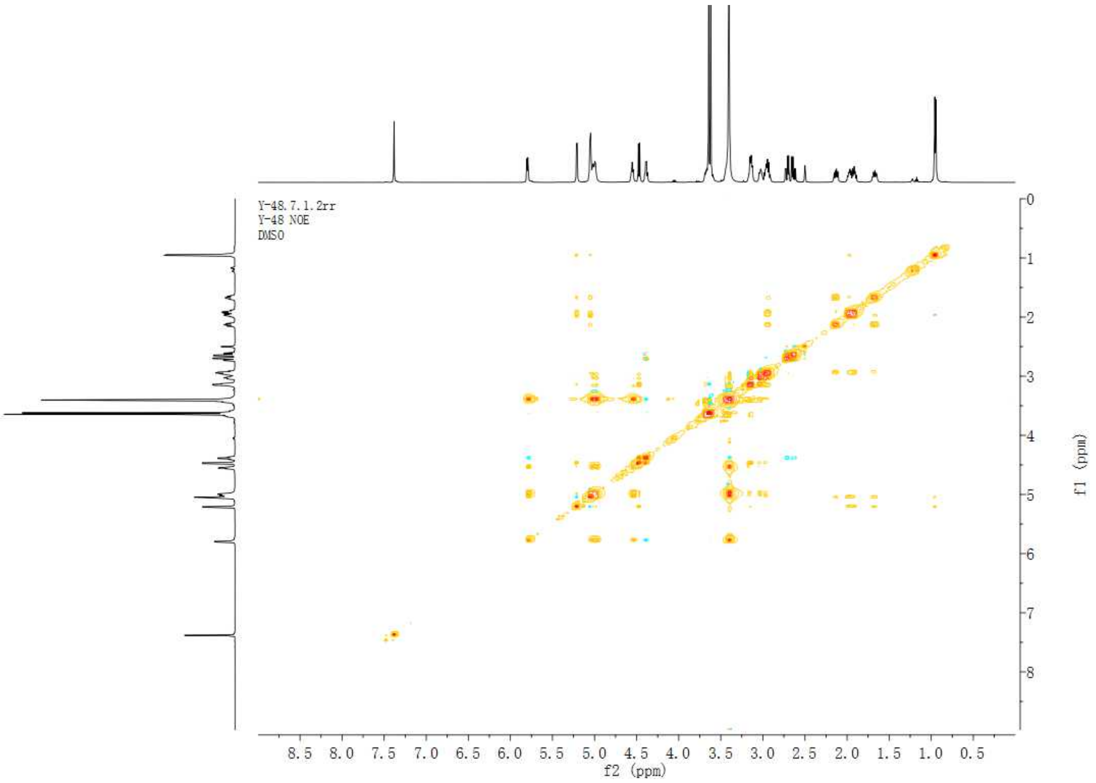

Figure S7. NOESY spectrum of compound **1**

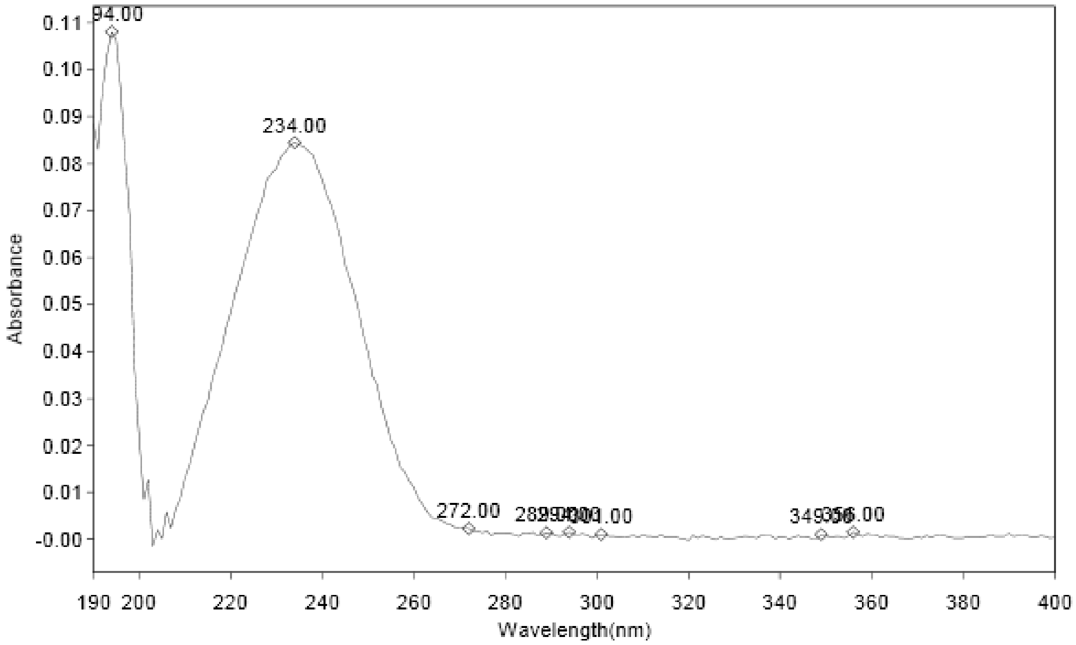

Results Table - Y-48-1.sre,Y-48-1,Cycle01

nm	A	Peak Pick Method	
194.00	.108	Find 8 Peaks Above -3.0000 A	
234.00	.084	Start Wavelength 190.00 nm	
272.00	.002	Stop Wavelength 400.00 nm	
289.00	.001	Sort By Wavelength	
294.00	.002	Sensitivity	Manual
301.00	.001	Rising Points	2
349.00	.001	Falling Points	2
356.00	.002	Min. Change	0.0000

Figure S8. UV spectrum of compound **1**

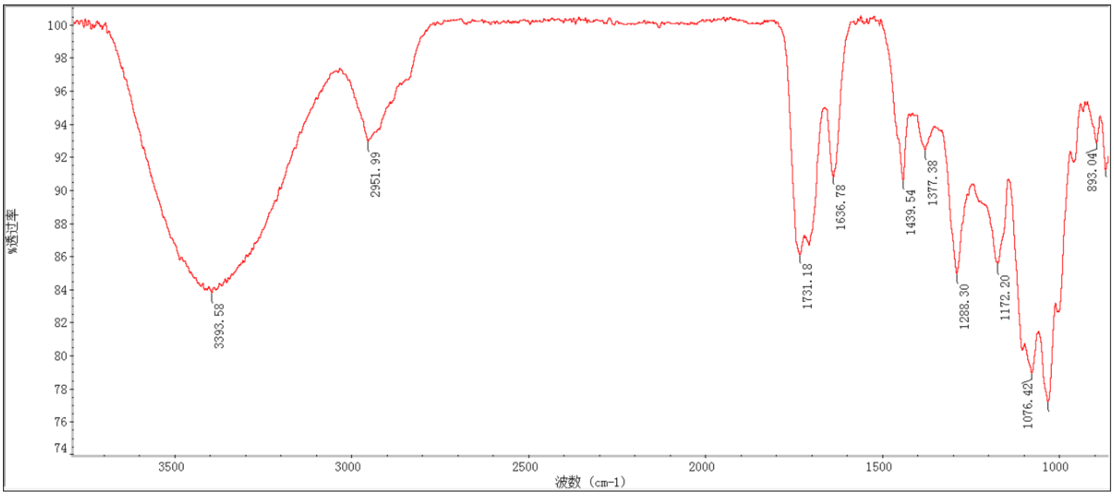

Figure S9. IR spectrum of compound **1**

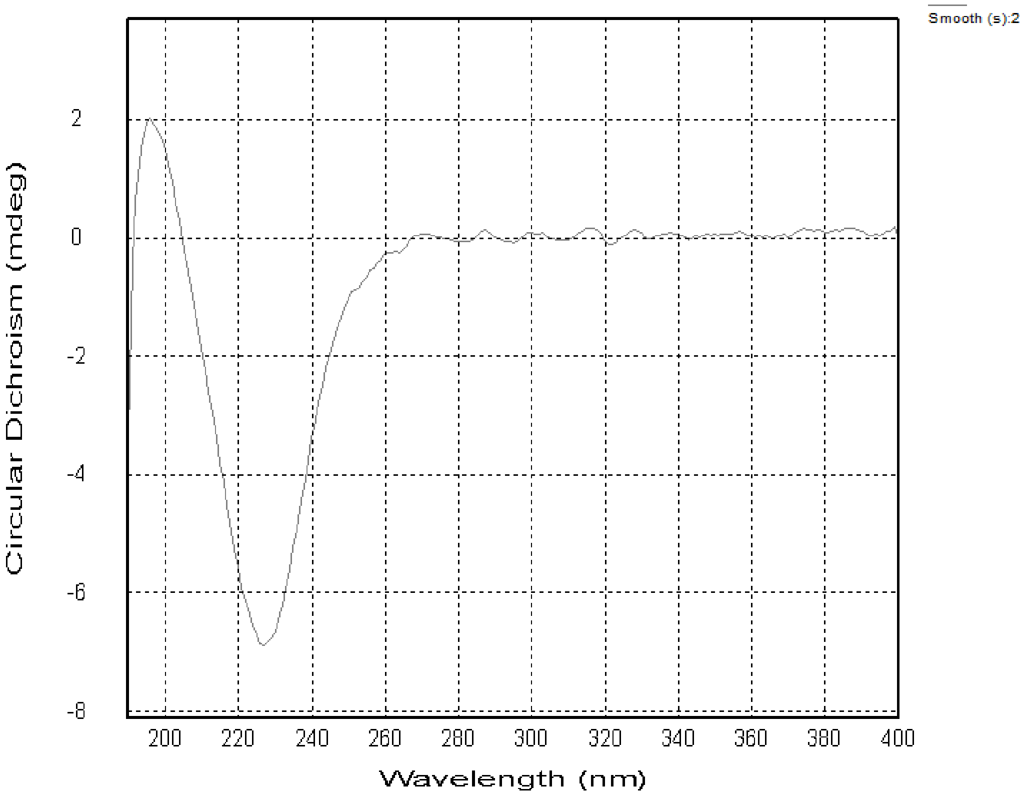

Figure S10. CD spectrum of compound **1**

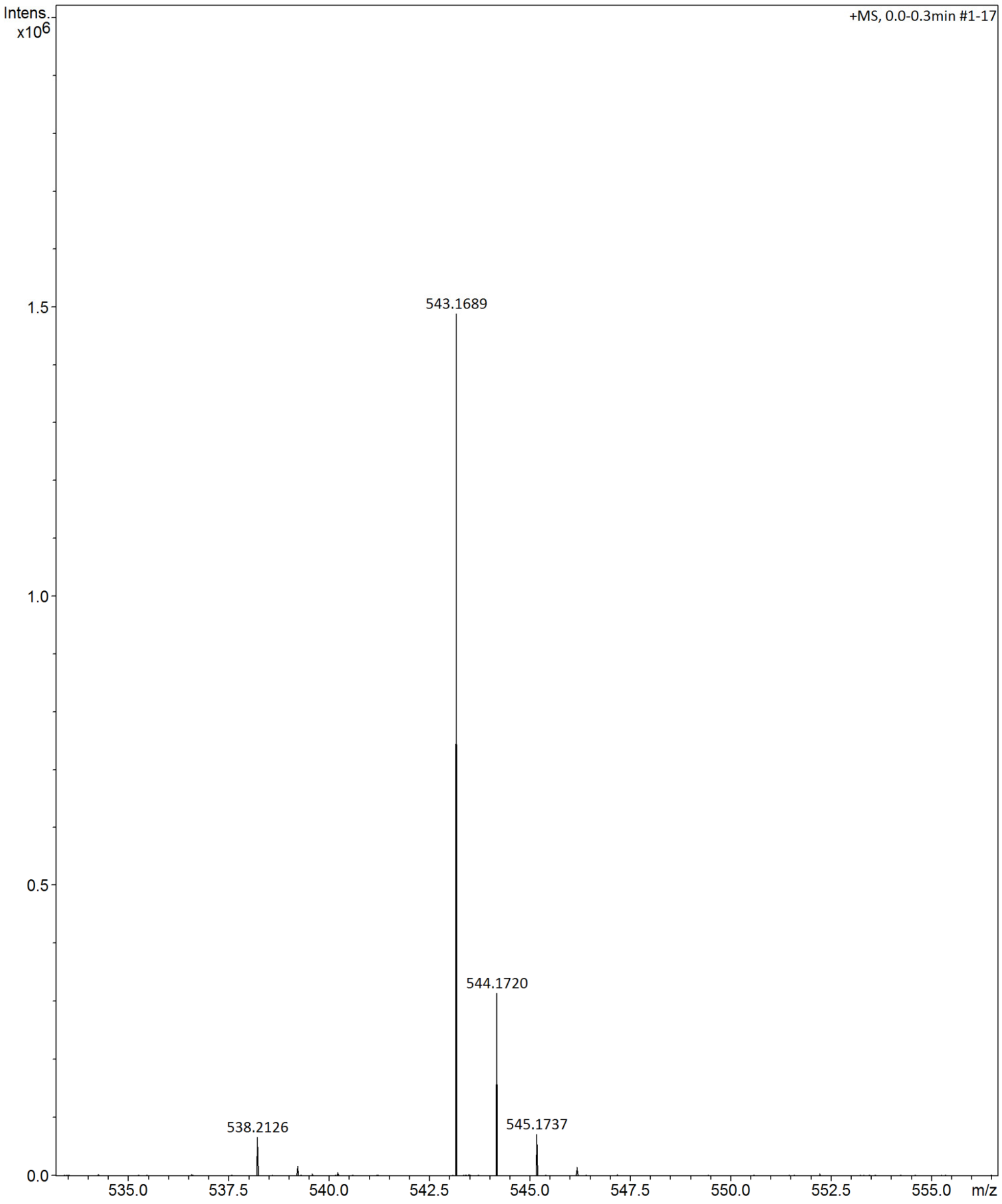

Figure S11. HRESIMS spectrum of compound **1**

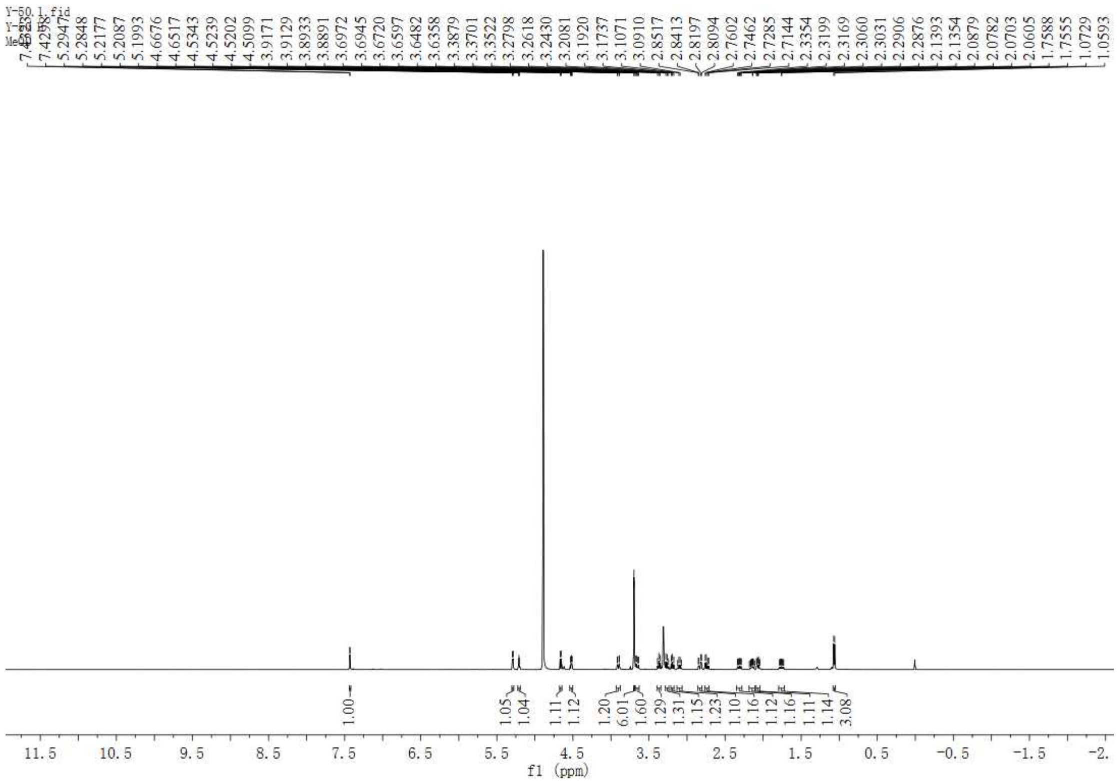

Figure S12. ^{1}H NMR spectrum of compound**2** (500 MHz, MeOD)

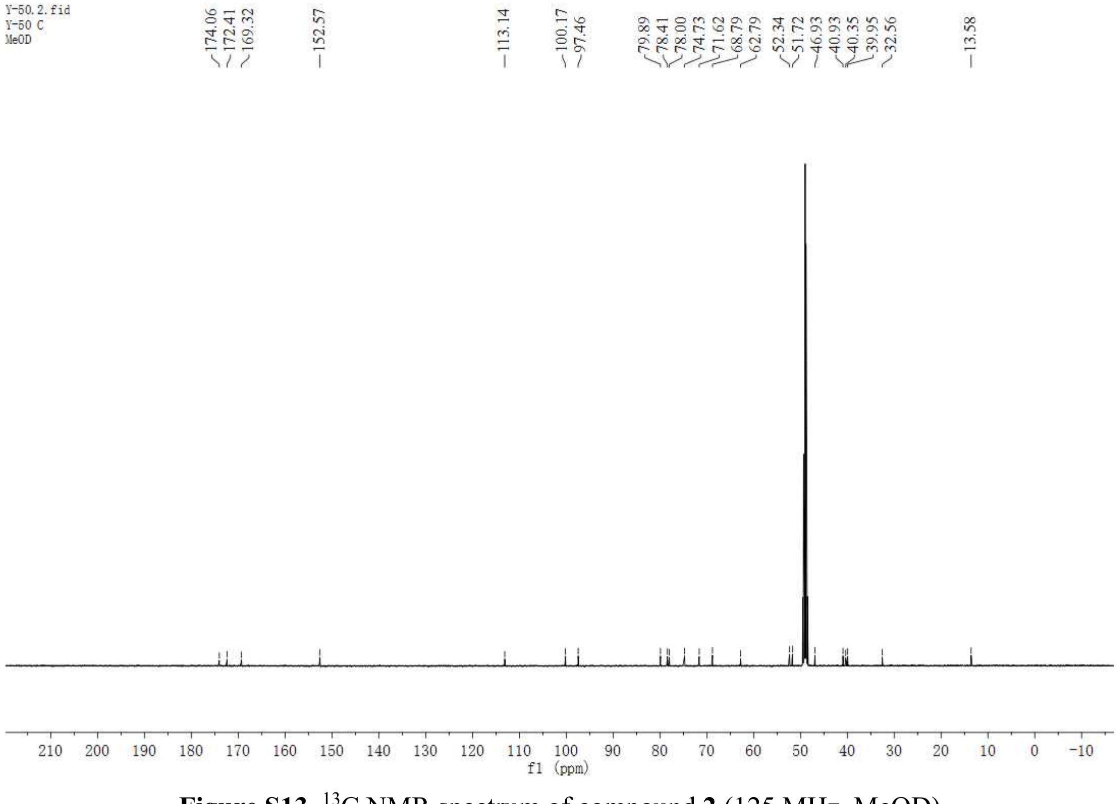

Figure S13. ^{13}C NMR spectrum of compound **2** (125 MHz, MeOD)

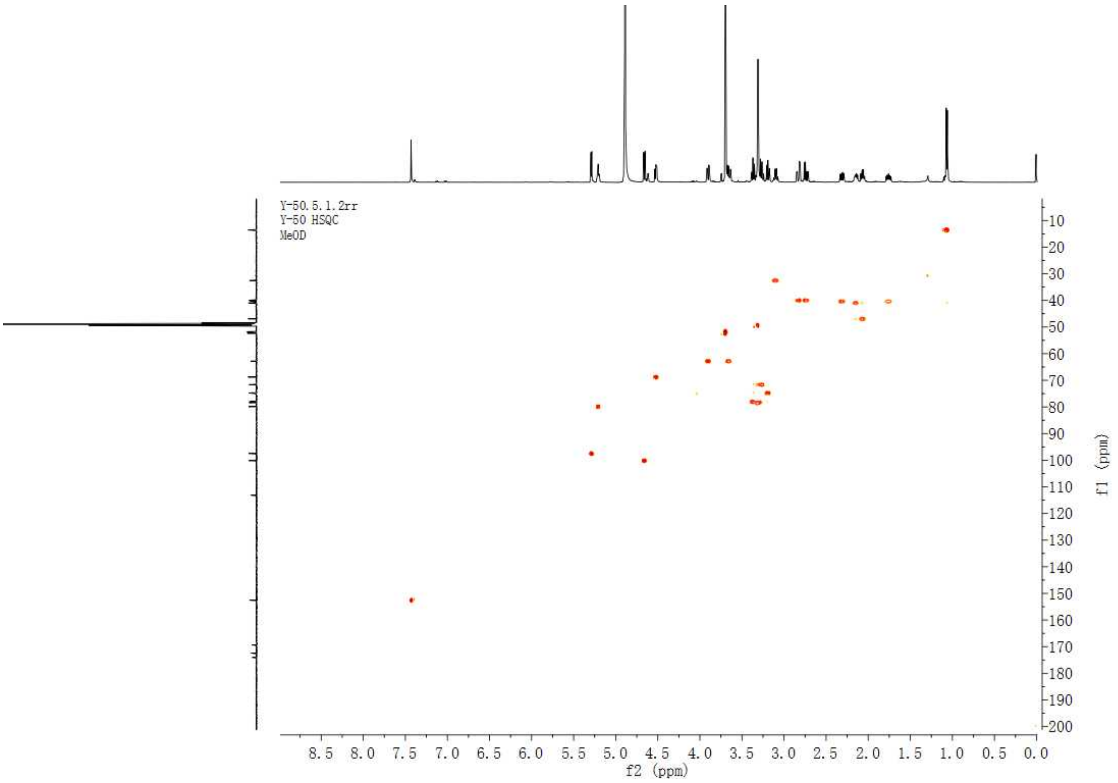

Figure S14. HSQC spectrum of compound **2**

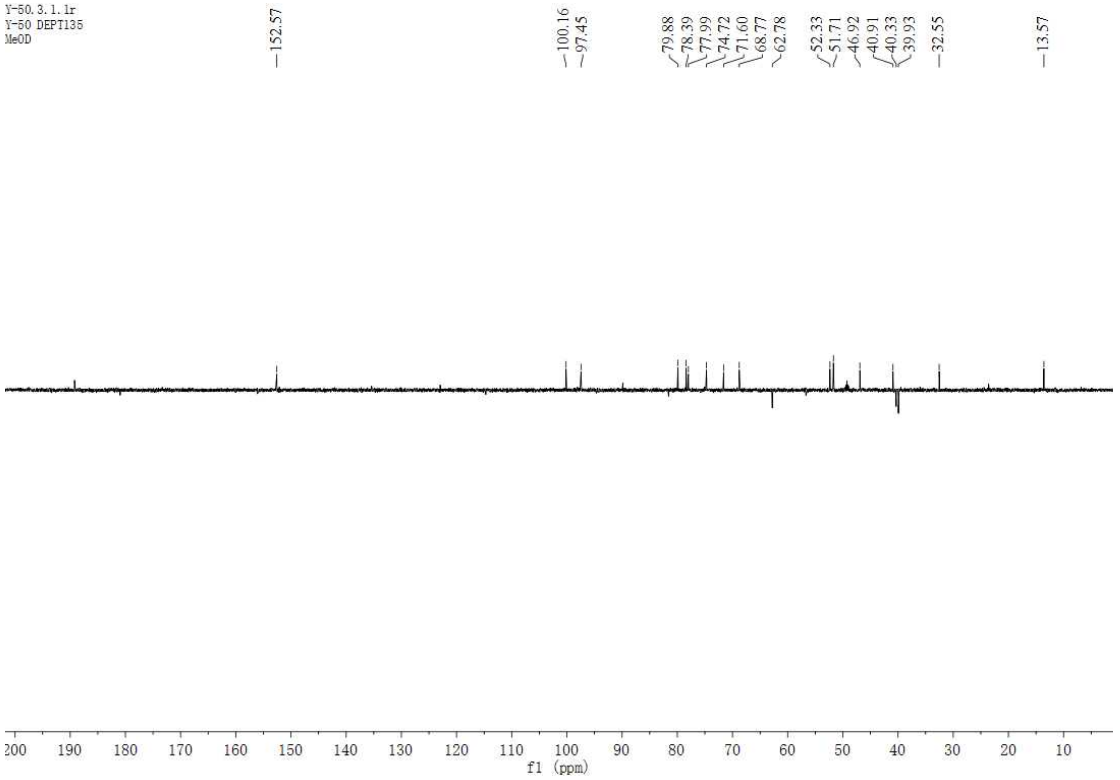

Figure S15. DEPT135 spectrum of compound **2**

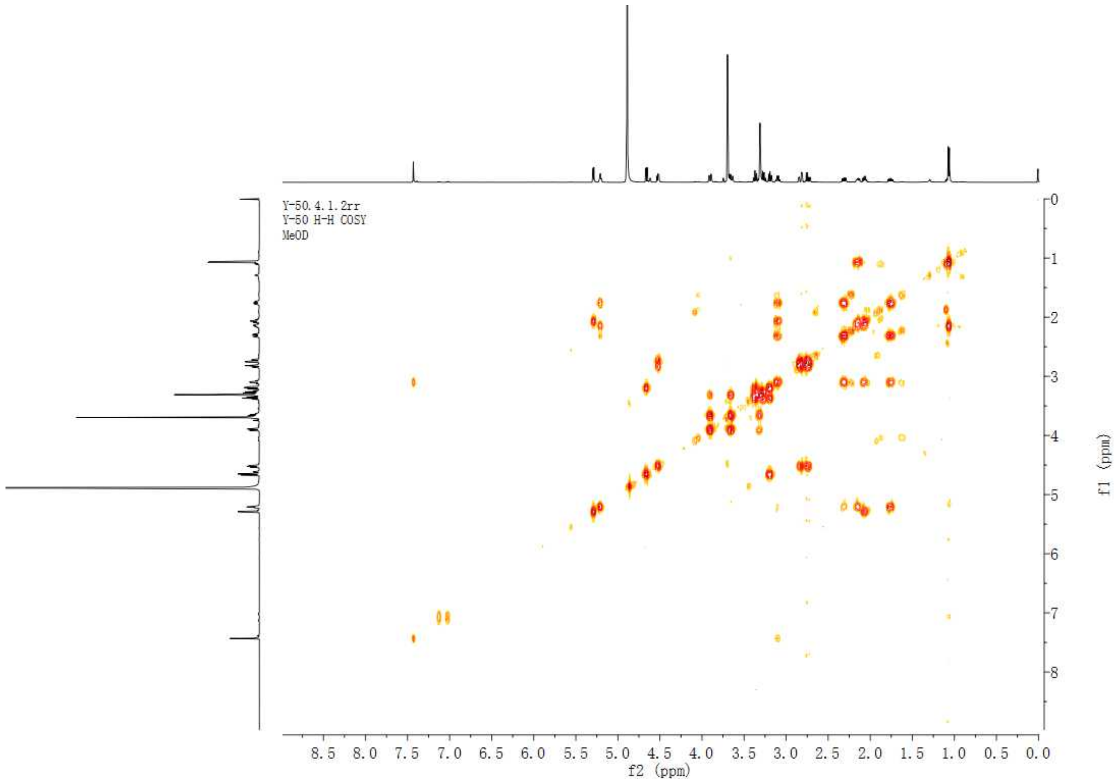

Figure S16. ^{1}H-^{1}H COSY spectrum of compound **2**

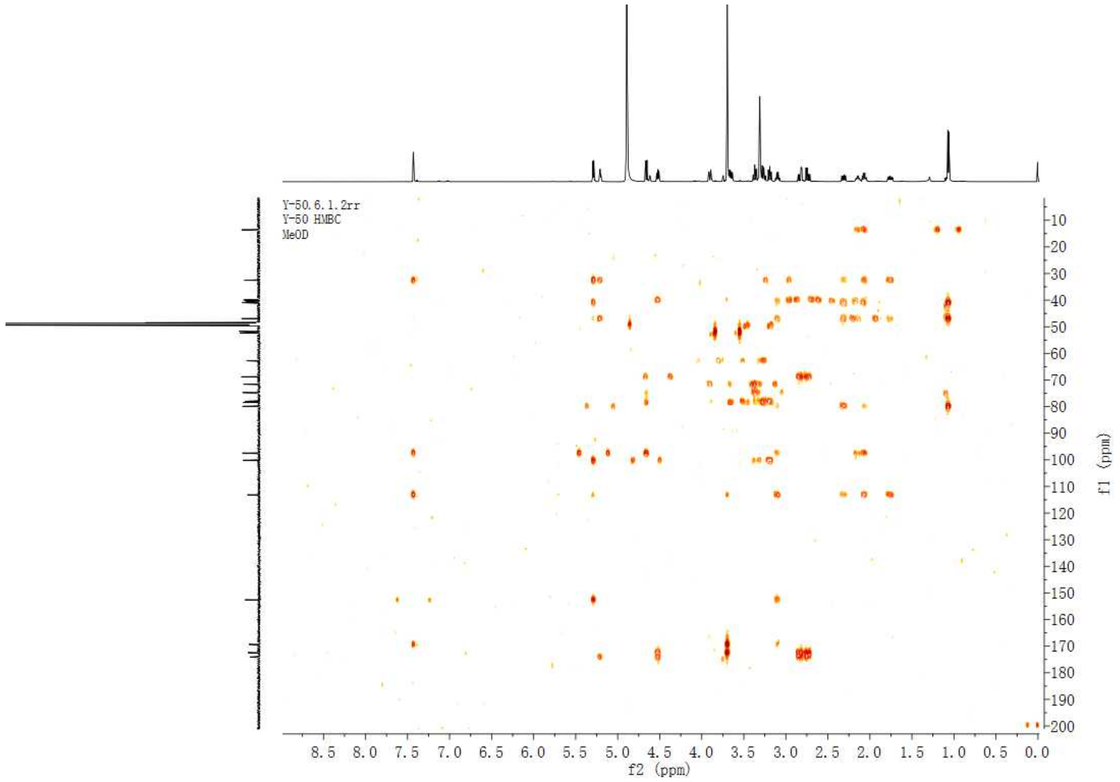

Figure S17. HMBC spectrum of compound **2**

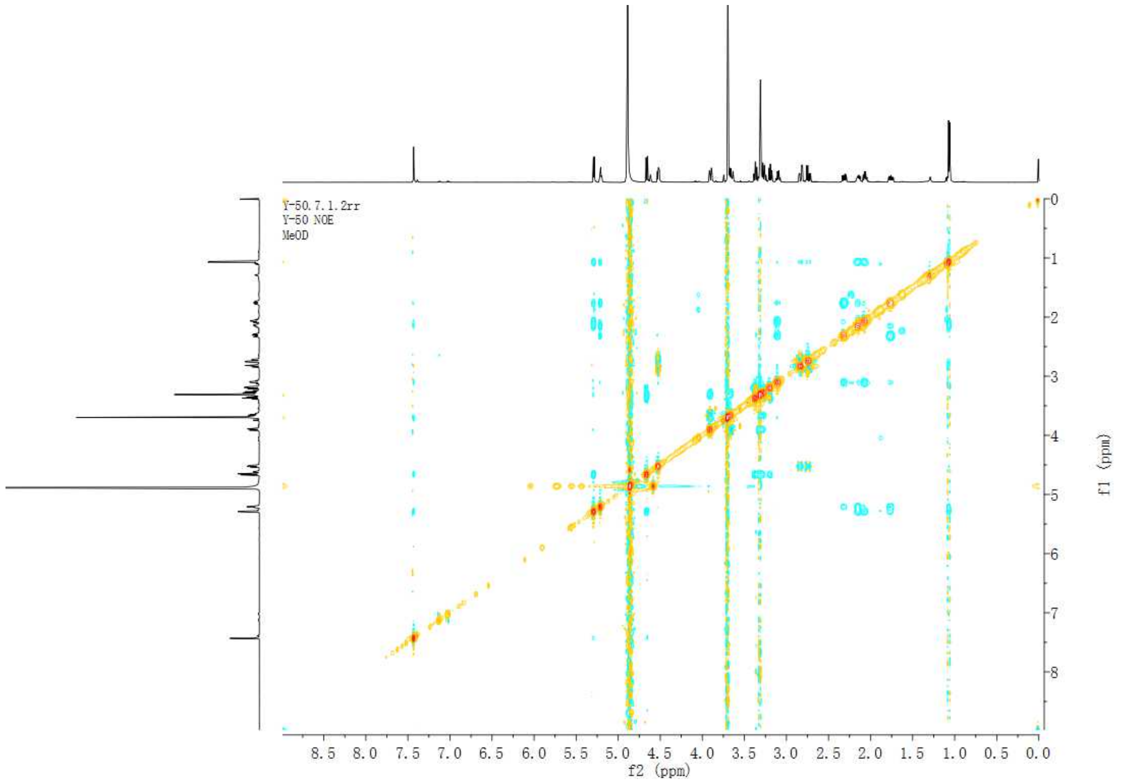

Figure S18. NOESY spectrum of compound **2**

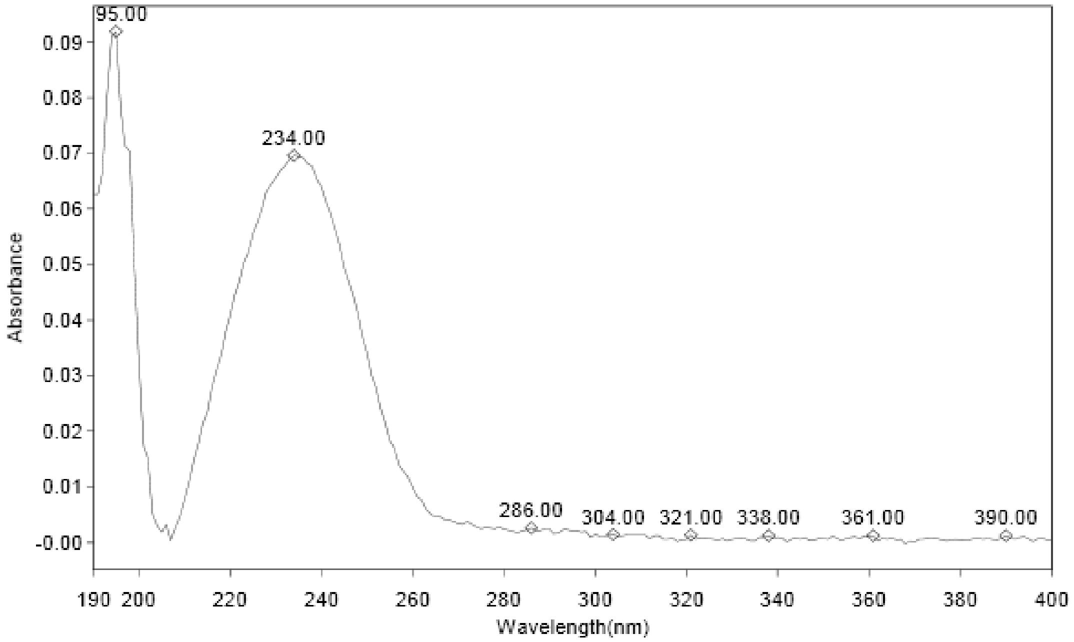

Results Table - Y-50-1.sre,Y-50-1,Cycle01

nm	A	Peak Pick Method	
195.00	.092	Find 8 Peaks Above -3.0000 A	
234.00	.070	Start Wavelength190.00 nm	
286.00	.003	Stop Wavelength400.00 nm	
304.00	.001	Sort By Wavelength	
321.00	.001	Sensitivity	Manual
338.00	.001	Rising Points	2
361.00	.001	Falling Points	2
390.00	.001	Min. Change	0.0000

Figure S19. UV spectrum of compound **2**

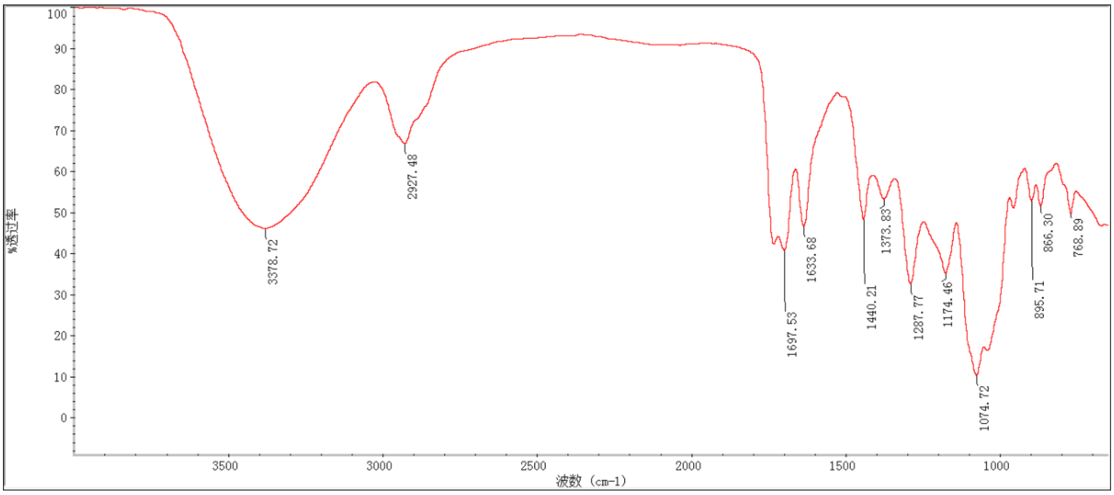

Figure S20. IR spectrum of compound **2**

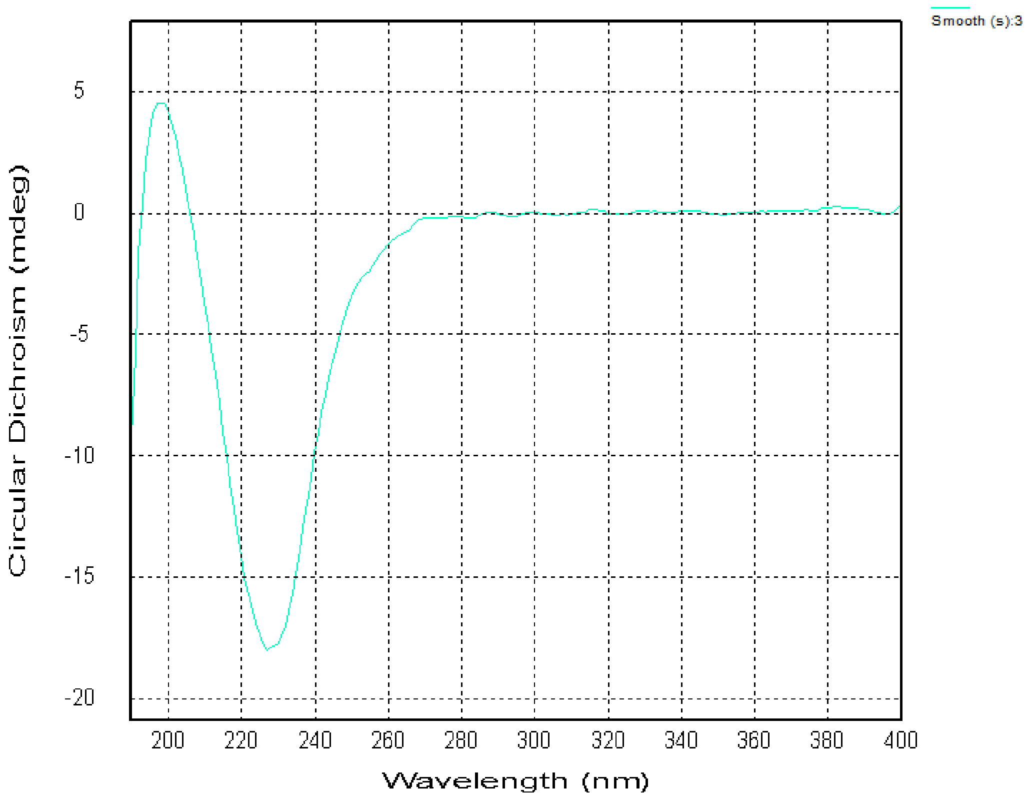

Figure S21. CD spectrum of compound **2**

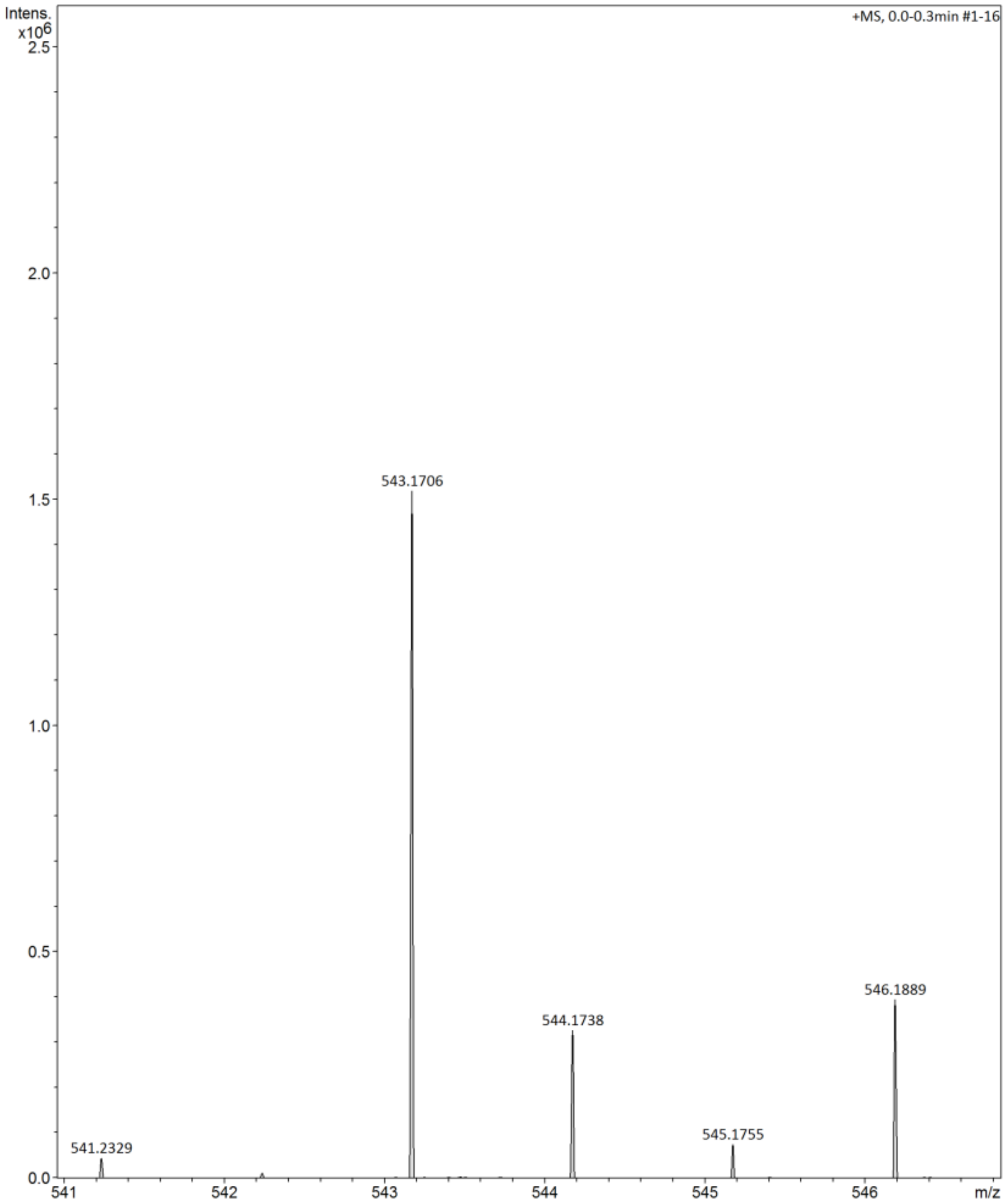

Figure S22. HRESIMS spectrum of compound **2**

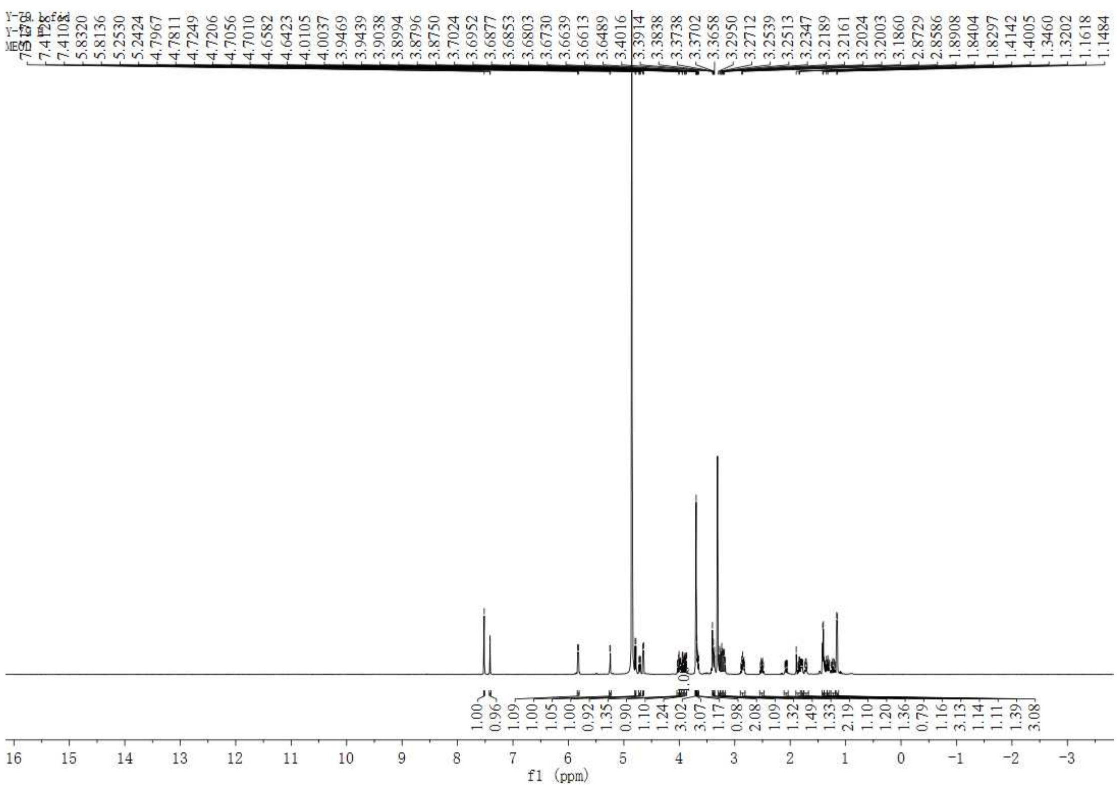

Figure S23. ^{1}H NMR spectrum of compound **3** (500 MHz, MeOD)

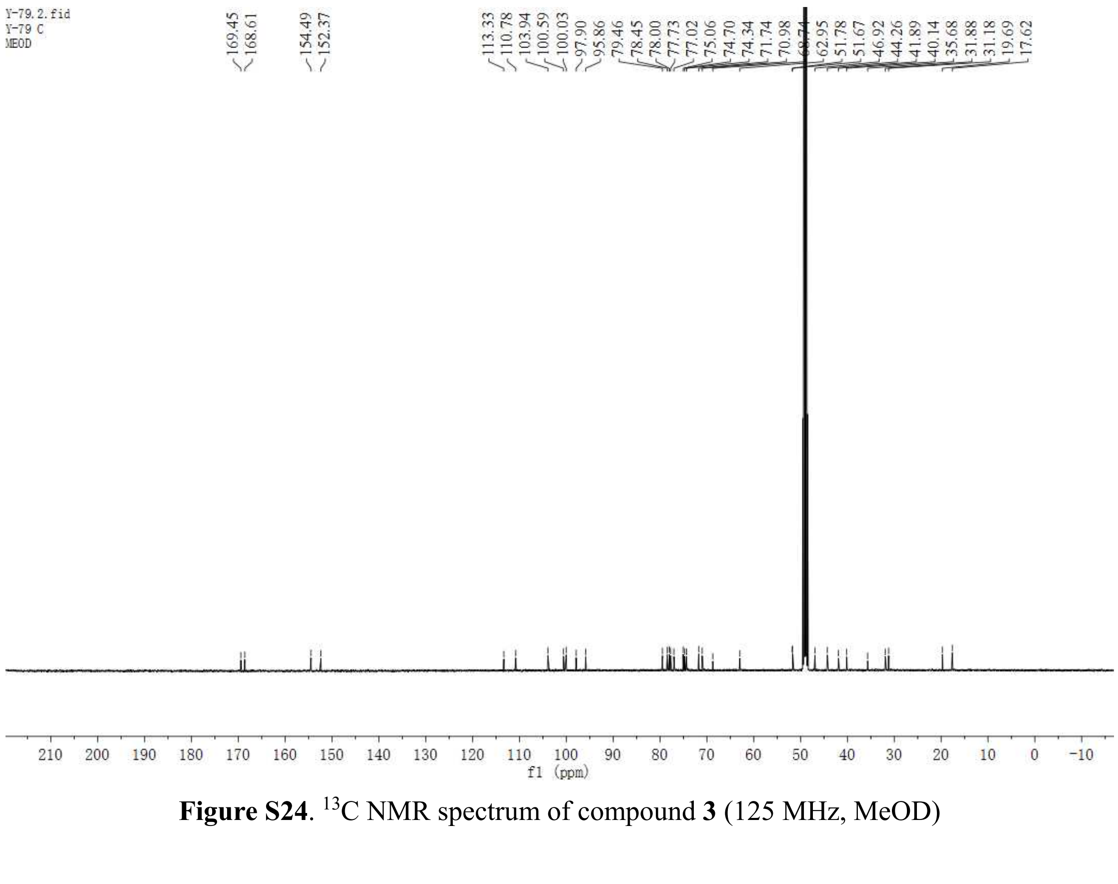

Figure S24. ^{13}C NMR spectrum of compound **3** (125 MHz, MeOD)

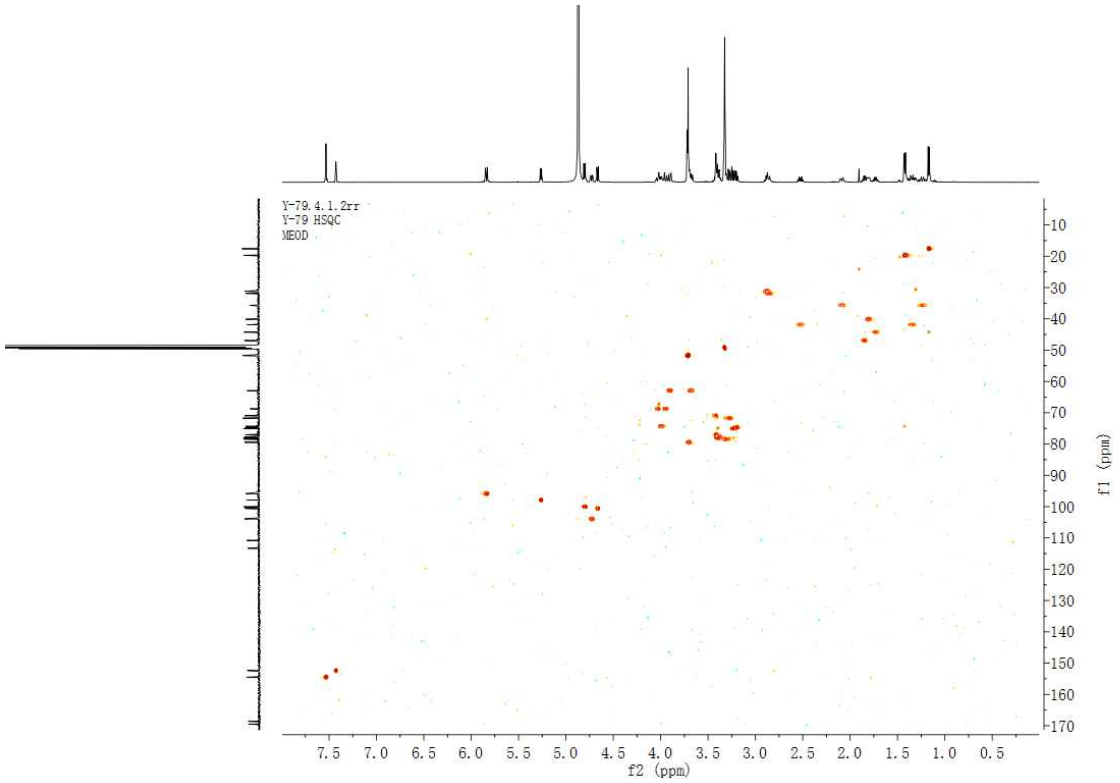

Figure S25. HSQC spectrum of compound **3**

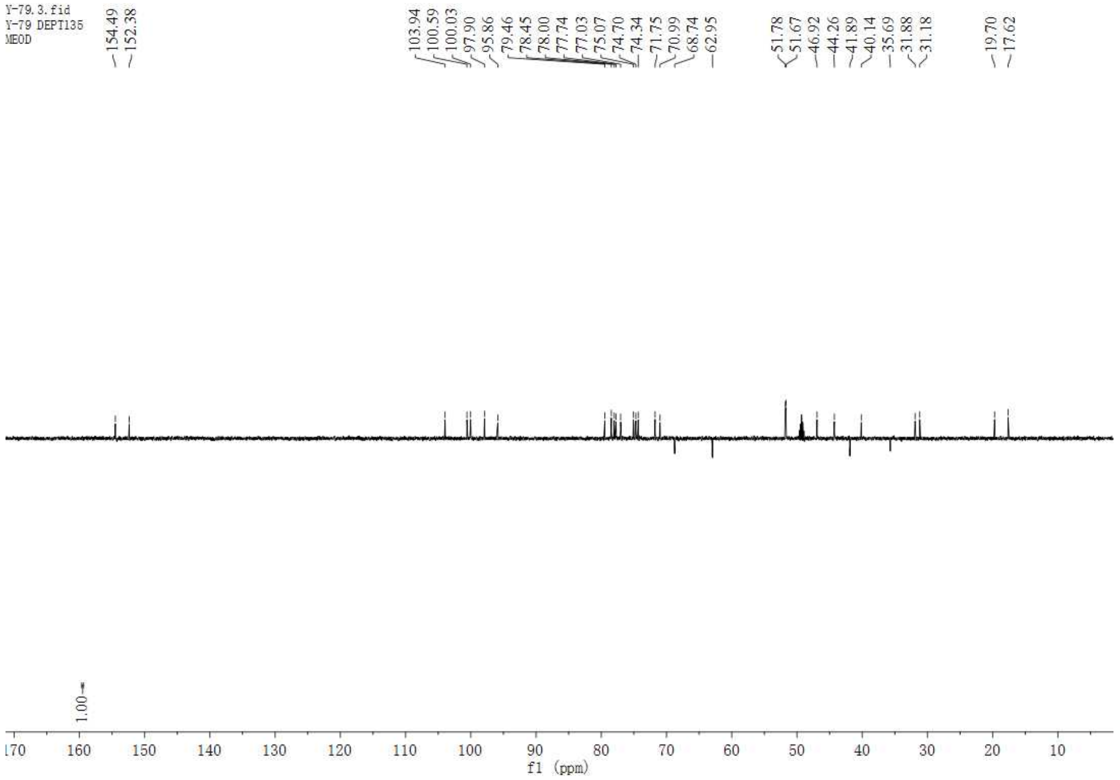

Figure S26. DEPT135 spectrum of compound **3**

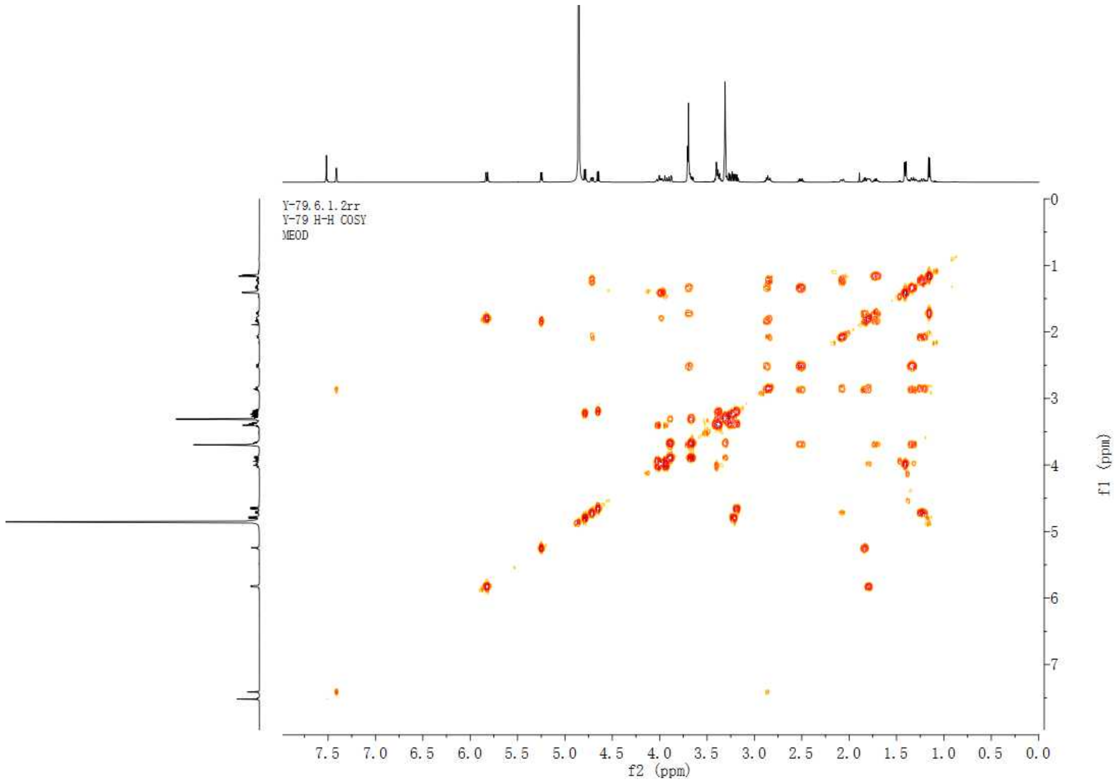

Figure S27. ^{1}H-^{1}H COSY spectrum of compound **3**

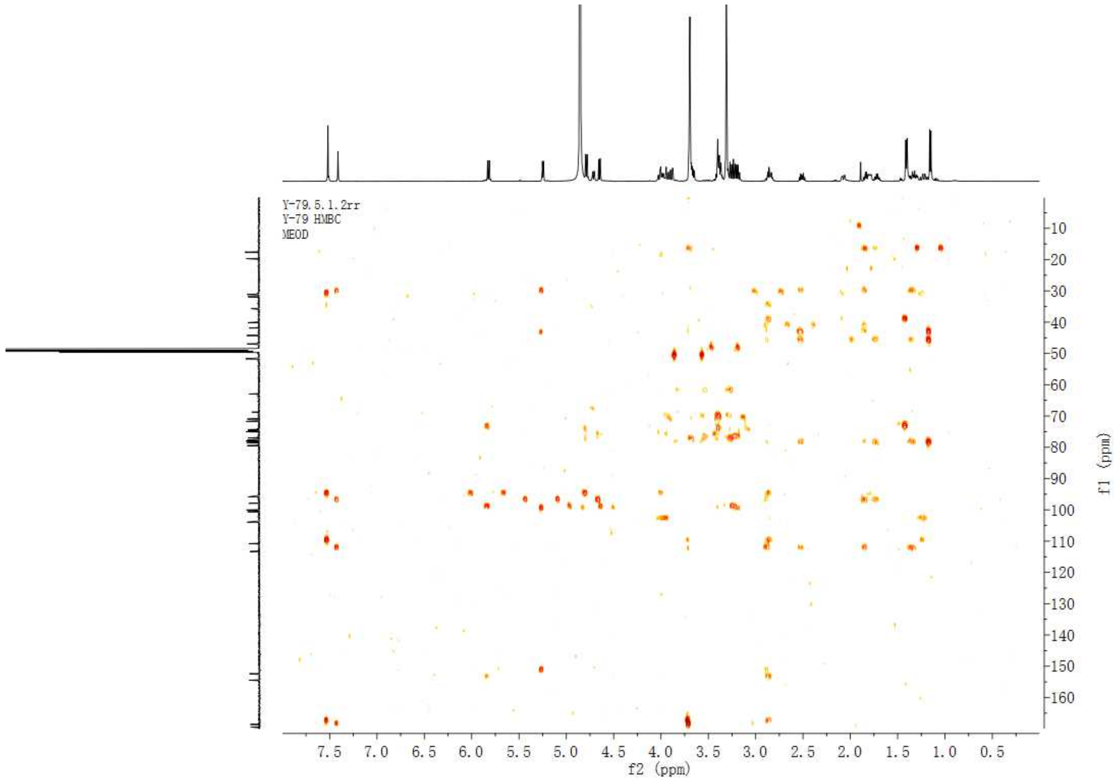

Figure S28. HMBC spectrum of compound **3**

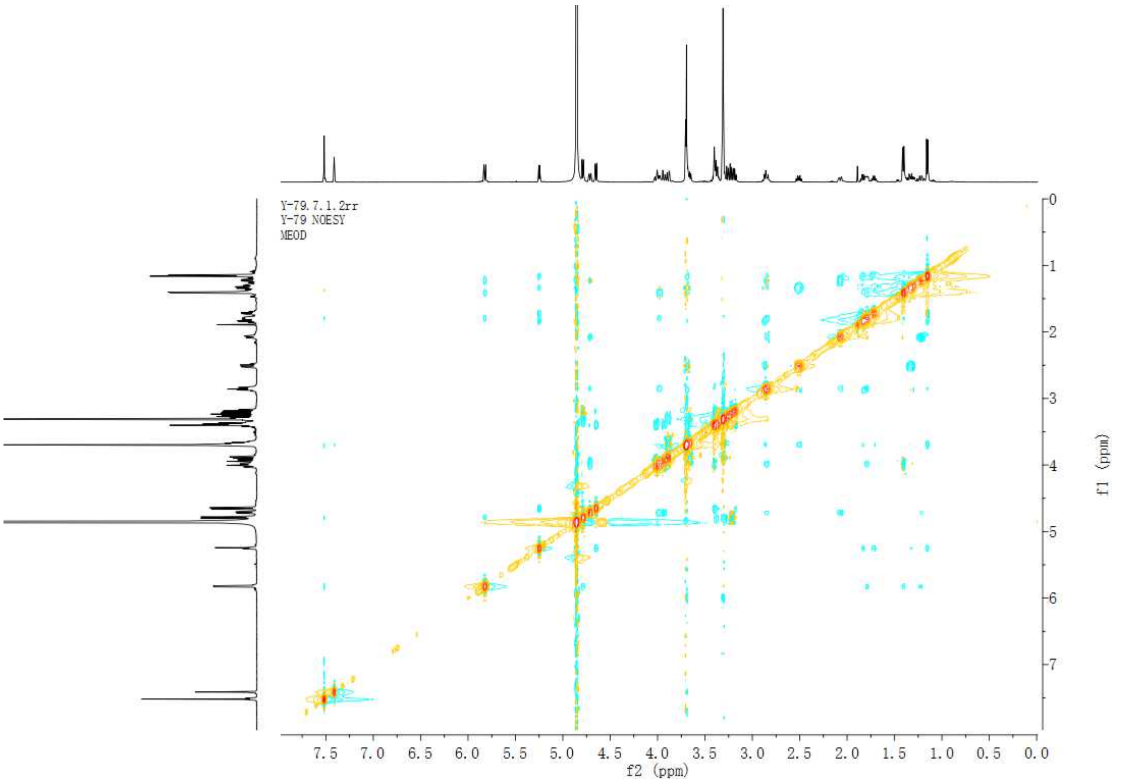

Figure S29. NOESY spectrum of compound **3**

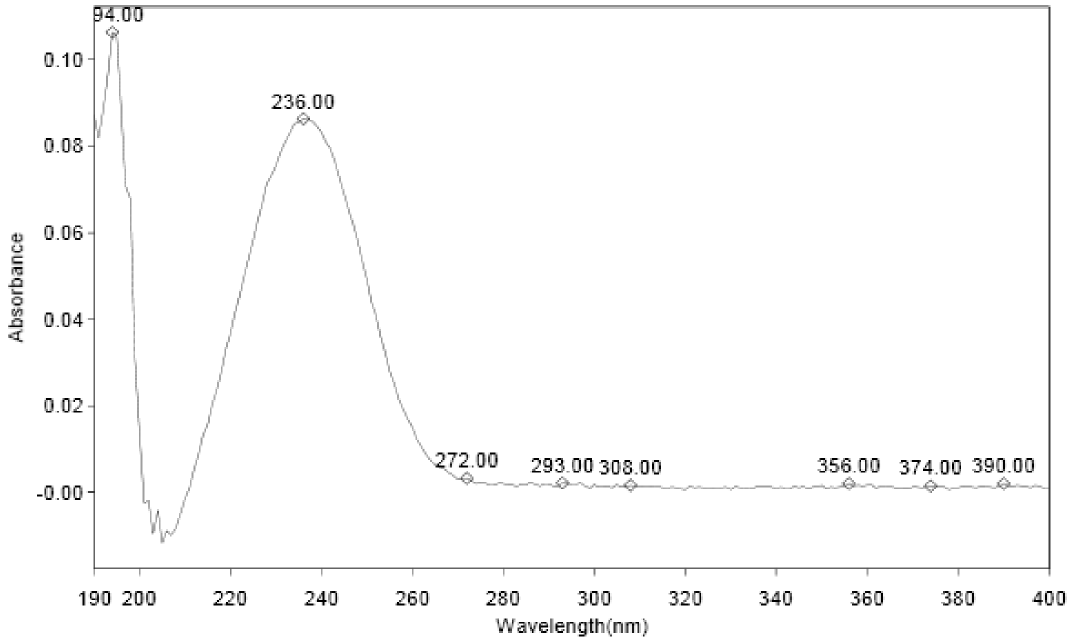

Results Table - Y-79-1.sre,Y-79-1,Cycle01

nm	A	**Peak Pick Method**	
194.00	.106	Find 8 Peaks Above -3.0000 A	
236.00	.086	Start Wavelength190.00 nm	
272.00	.003	Stop Wavelength400.00 nm	
293.00	.002	Sort By Wavelength	
308.00	.002	Sensitivity	Manual
356.00	.002	Rising Points	2
374.00	.002	Falling Points	2
390.00	.002	Min. Change	0.0000

Figure S30. UV spectrum of compound **3**

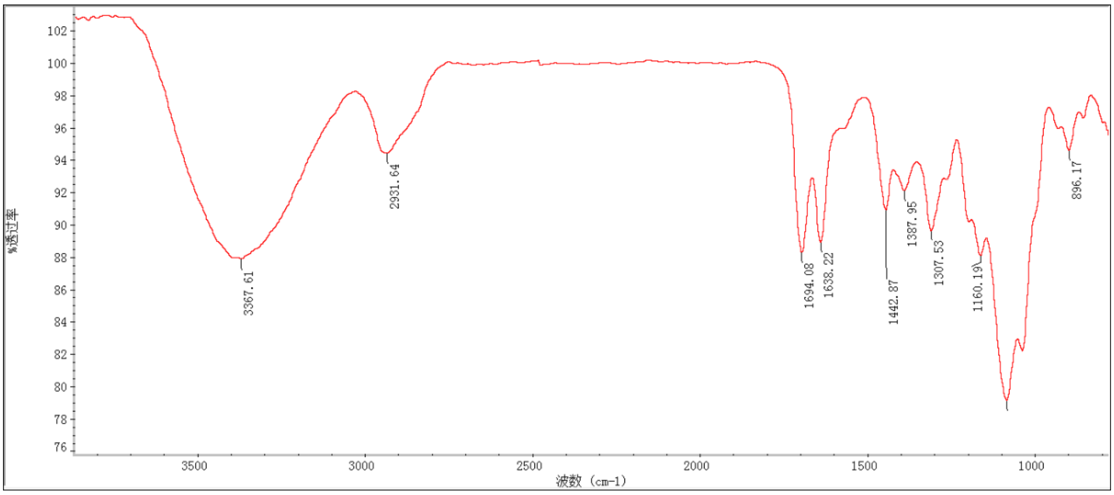

Figure S31. IR spectrum of compound **3**

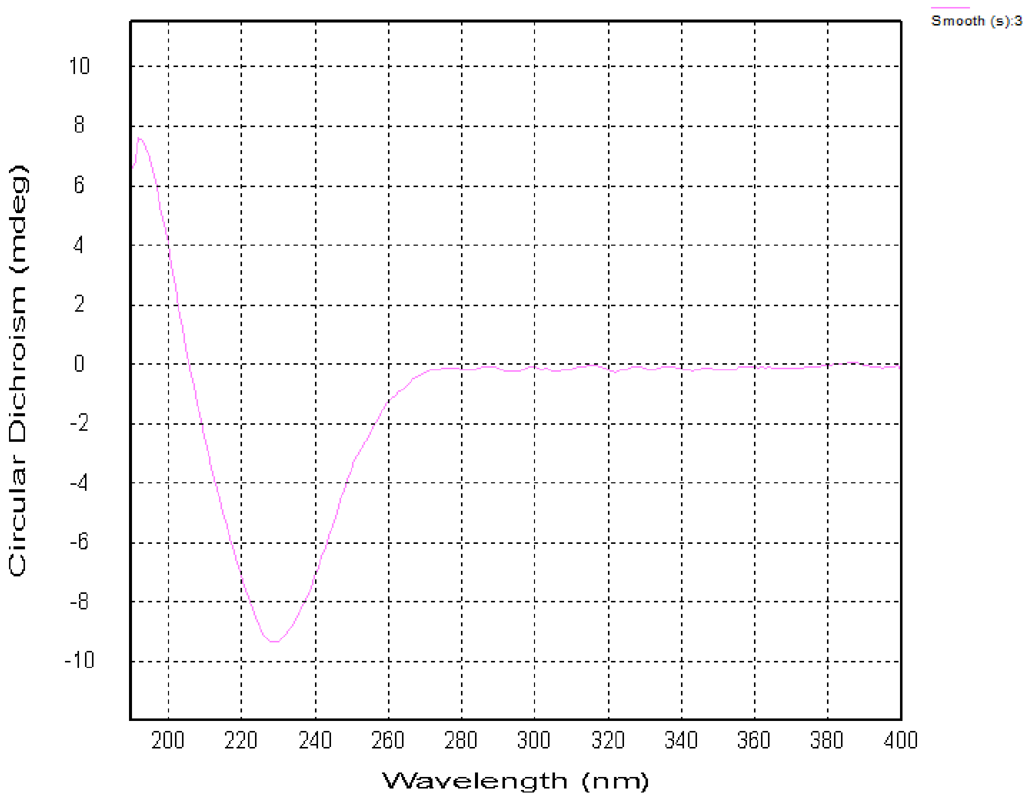

Figure S32. CD spectrum of compound **3**

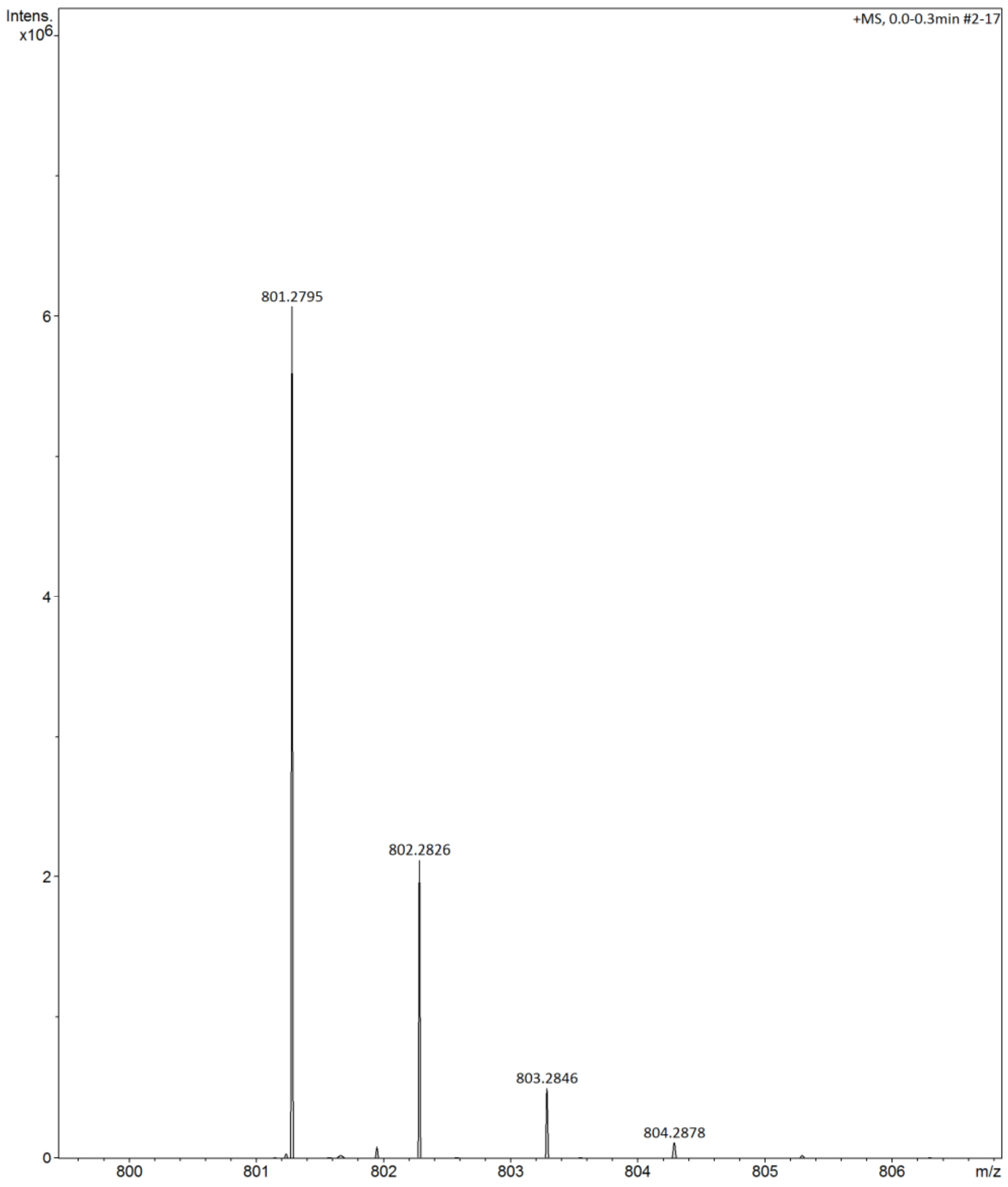

Figure S33. HRESIMS spectrum of compound **3**

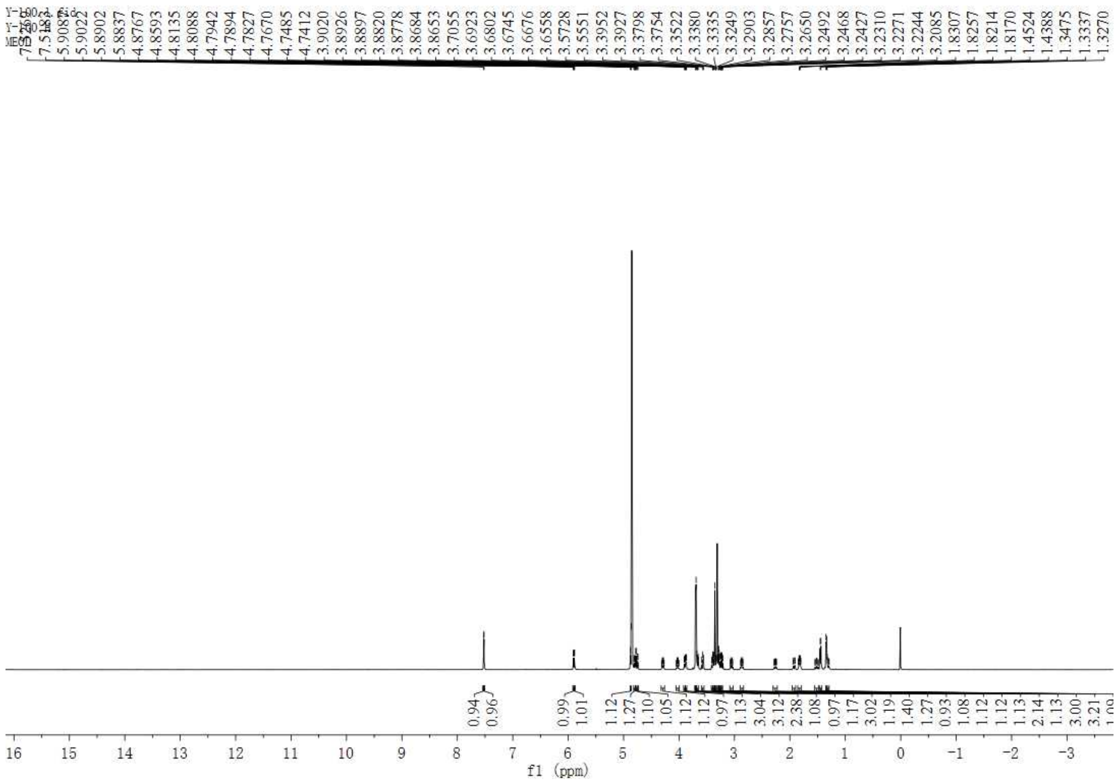

Figure S34. ^{1}H NMR spectrum of compound **4** (500 MHz, MeOD)

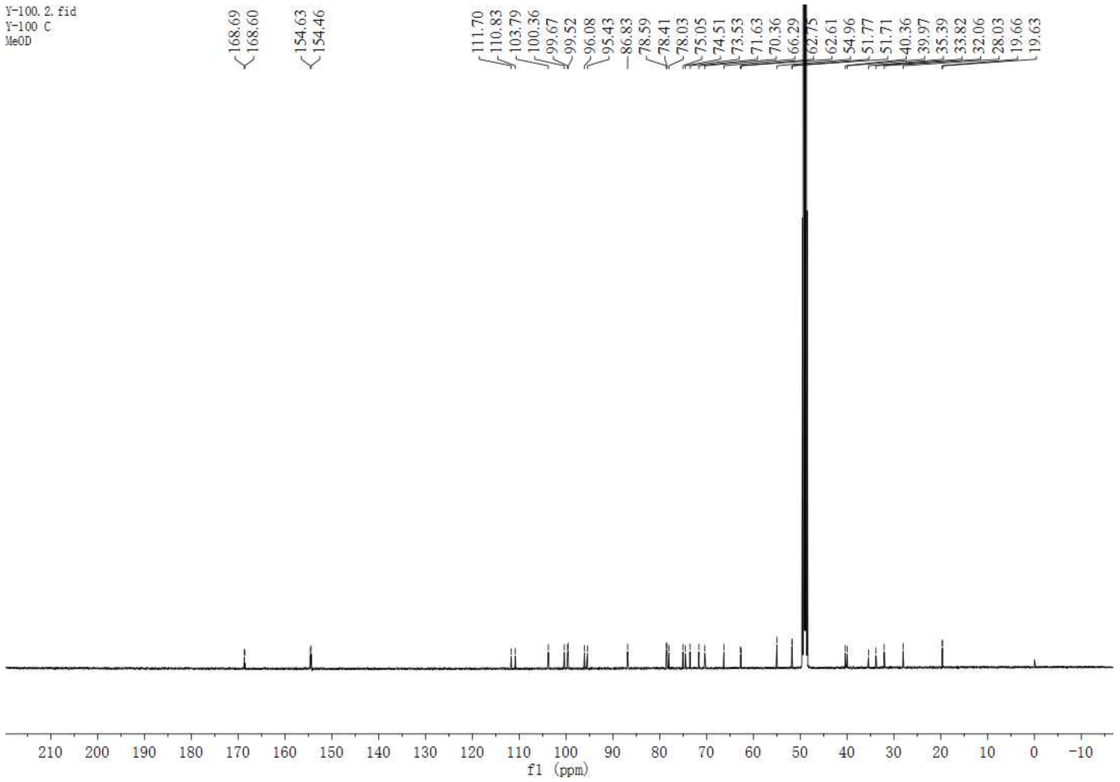

Figure S35. ^{13}C NMR spectrum of compound **4** (125 MHz, MeOD)

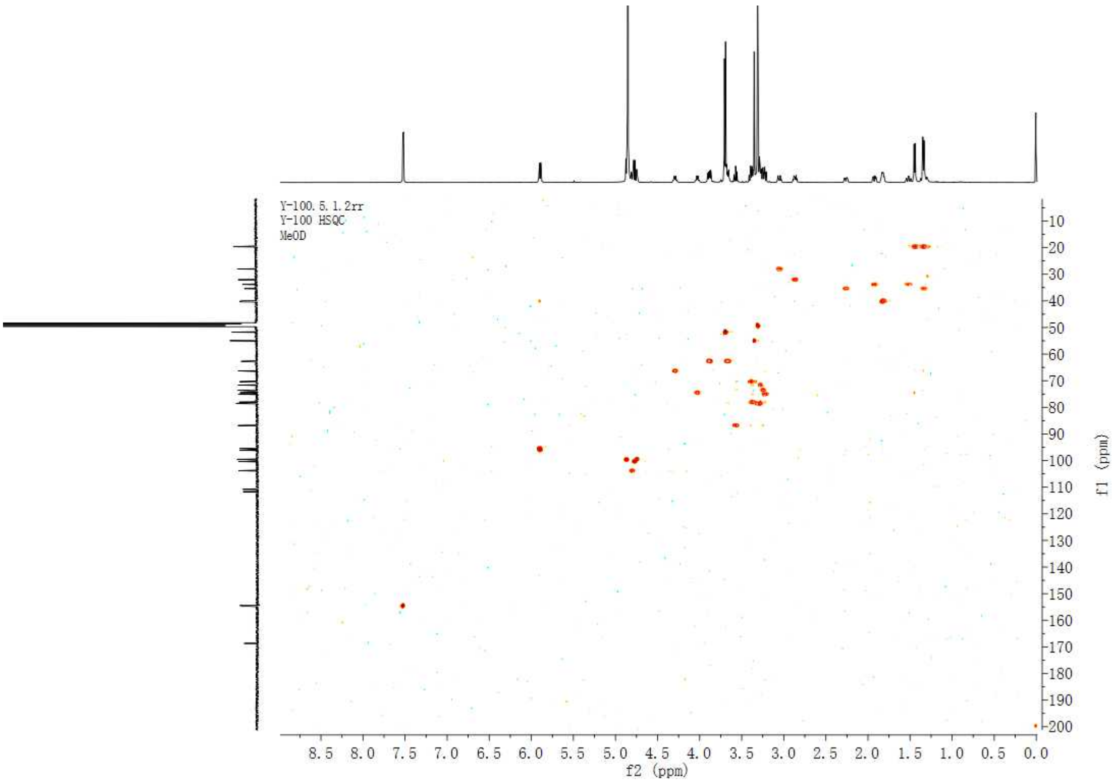

Figure S36. HSQC spectrum of compound **4**

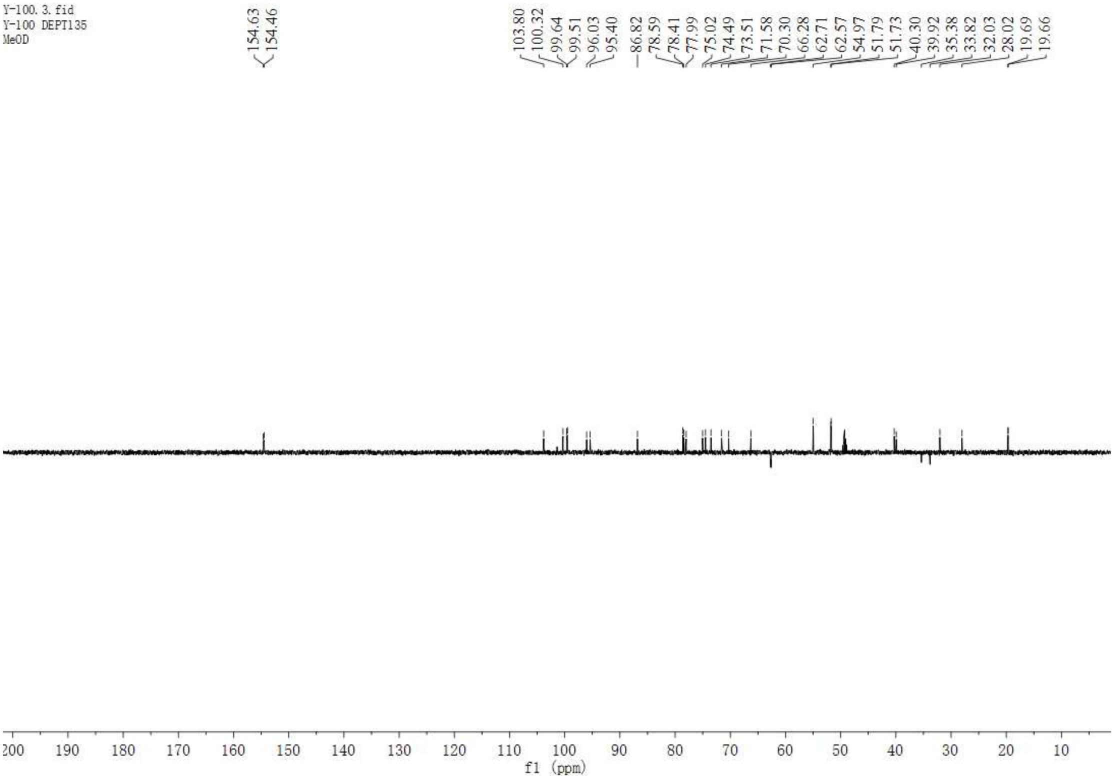

Figure S37. DEPT135 spectrum of compound **4**

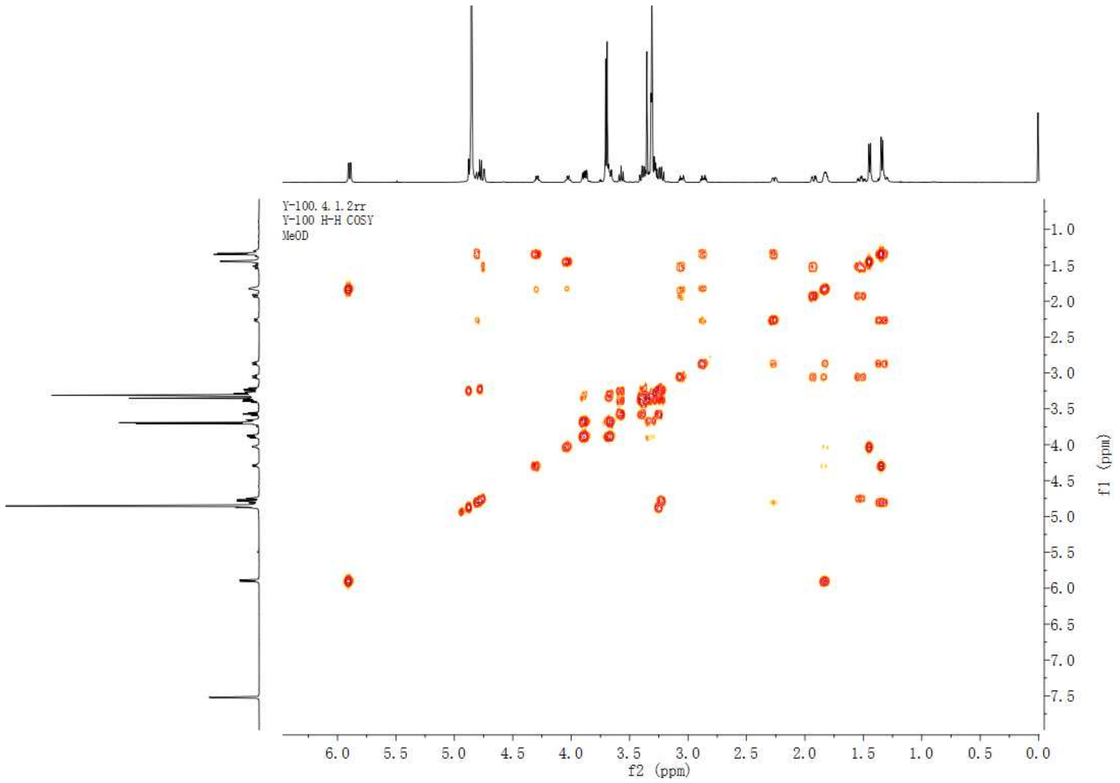

Figure S38. ^{1}H-^{1}H COSY spectrum of compound **4**

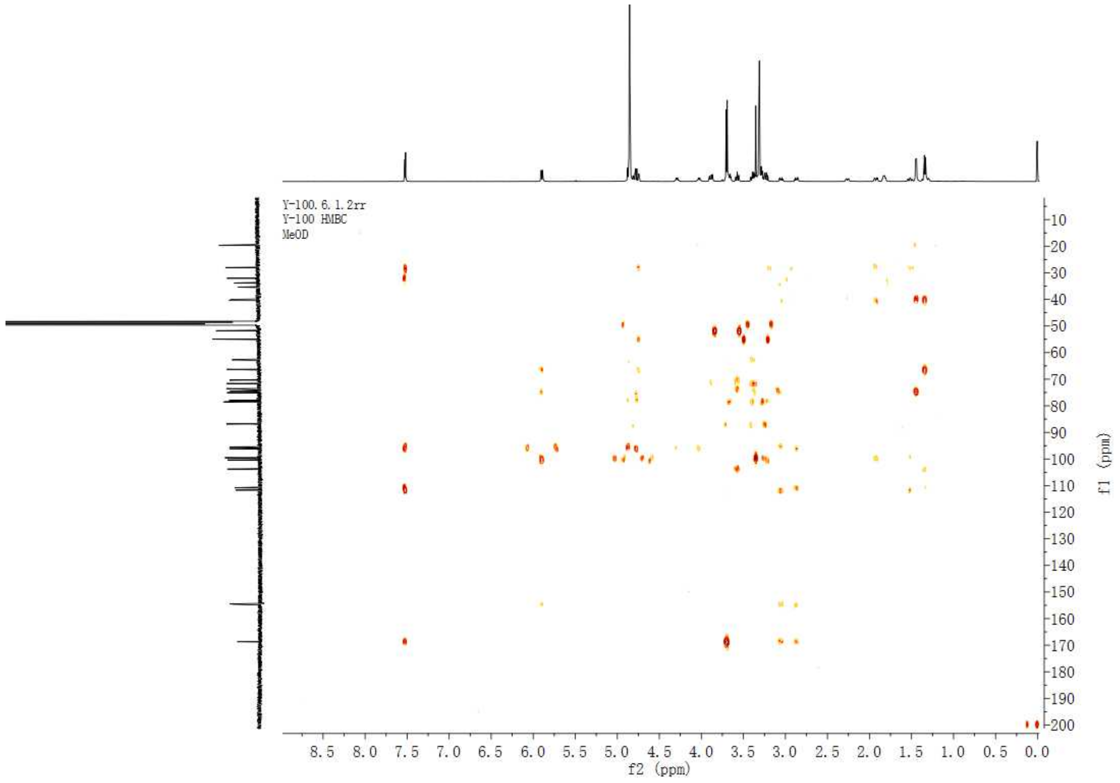

Figure S39. HMBC spectrum of compound **4**

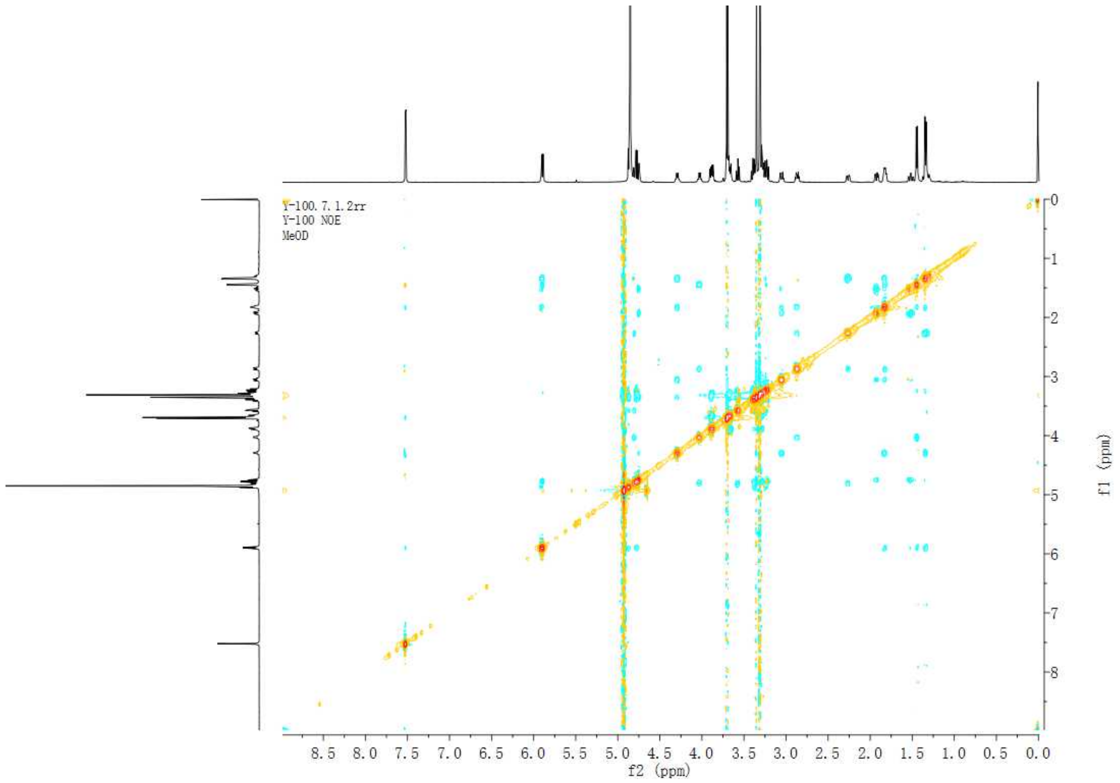

Figure S40. NOESY spectrum of compound **4**

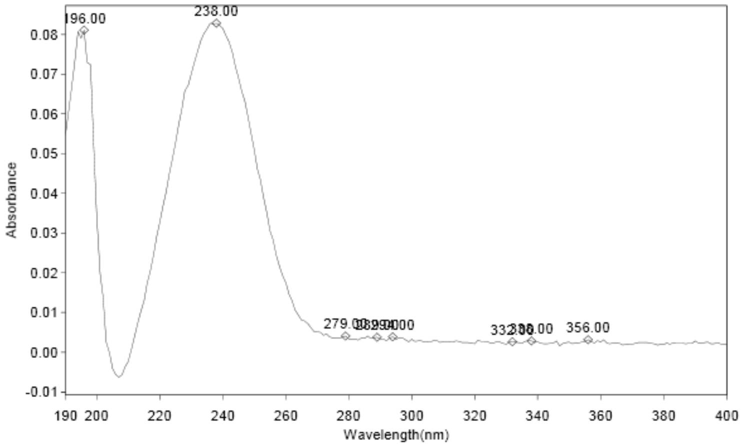

Results Table - Y-100-1.sre,Y-100-1,Cycle01

nm	A	Peak Pick Method	
196.00	.081	Find 8 Peaks Above -3.0000 A	
238.00	.083	Start Wavelength 190.00 nm	
279.00	.004	Stop Wavelength 400.00 nm	
289.00	.004	Sort By Wavelength	
294.00	.004	Sensitivity	Manual
332.00	.003	Rising Points	2
338.00	.003	Falling Points	2
356.00	.003	Min. Change	0.0000

Figure S41. UV spectrum of compound **4**

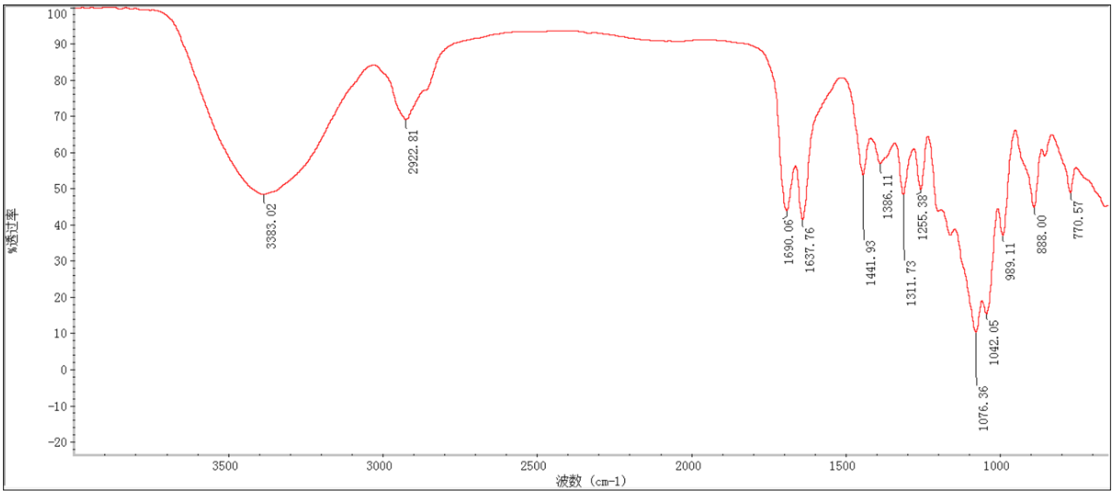

Figure S42. IR spectrum of compound **4**

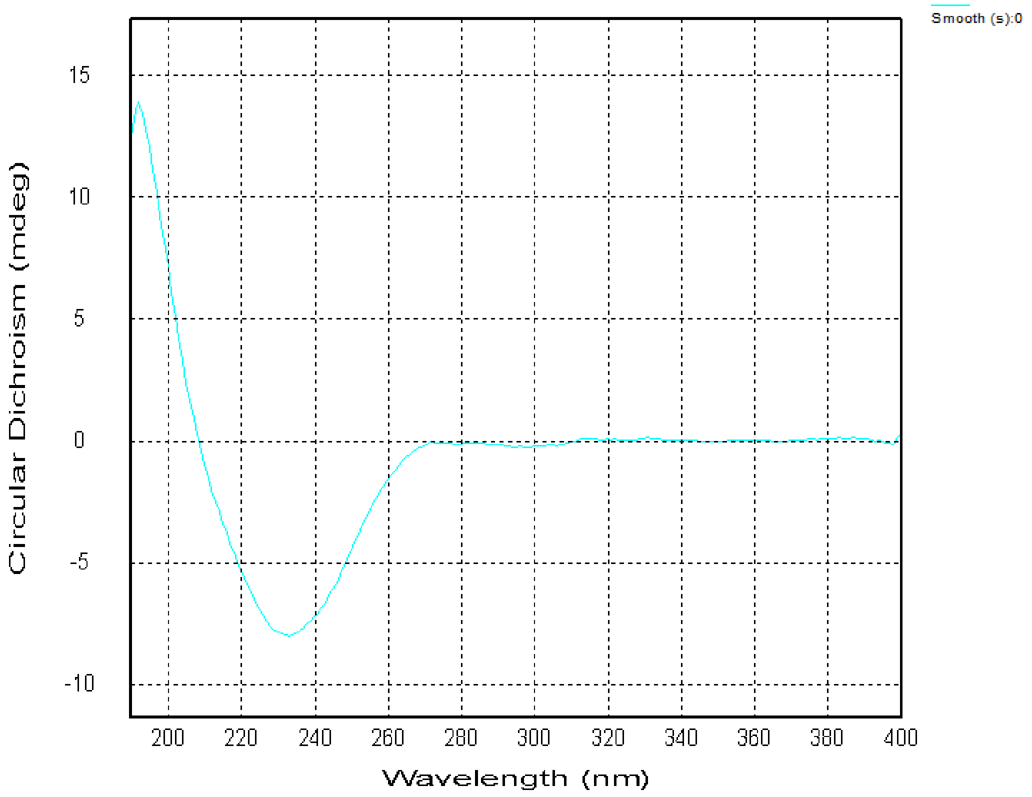

Figure S43. CD spectrum of compound **4**

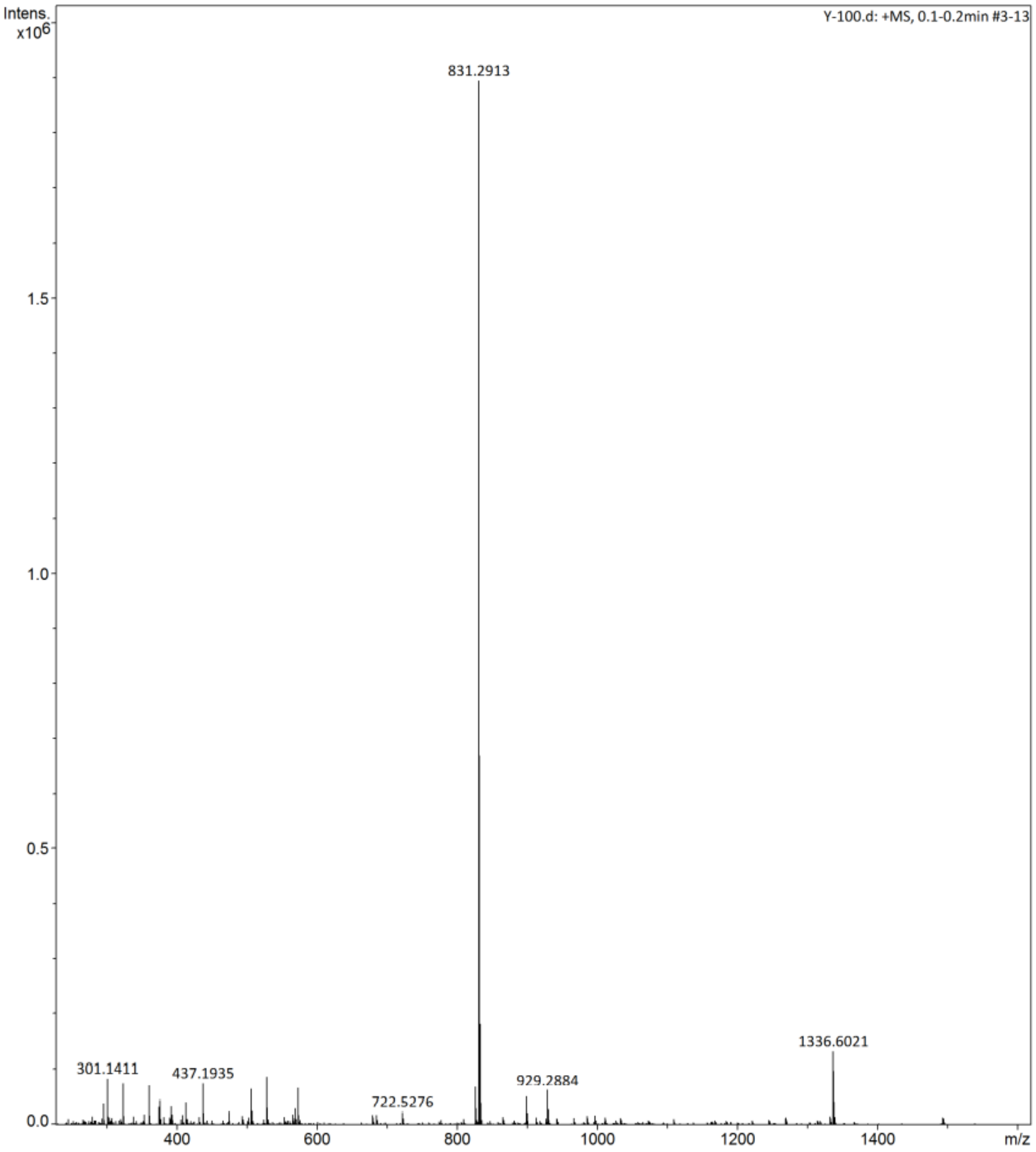

Figure S44. HRESIMS spectrum of compound **4**

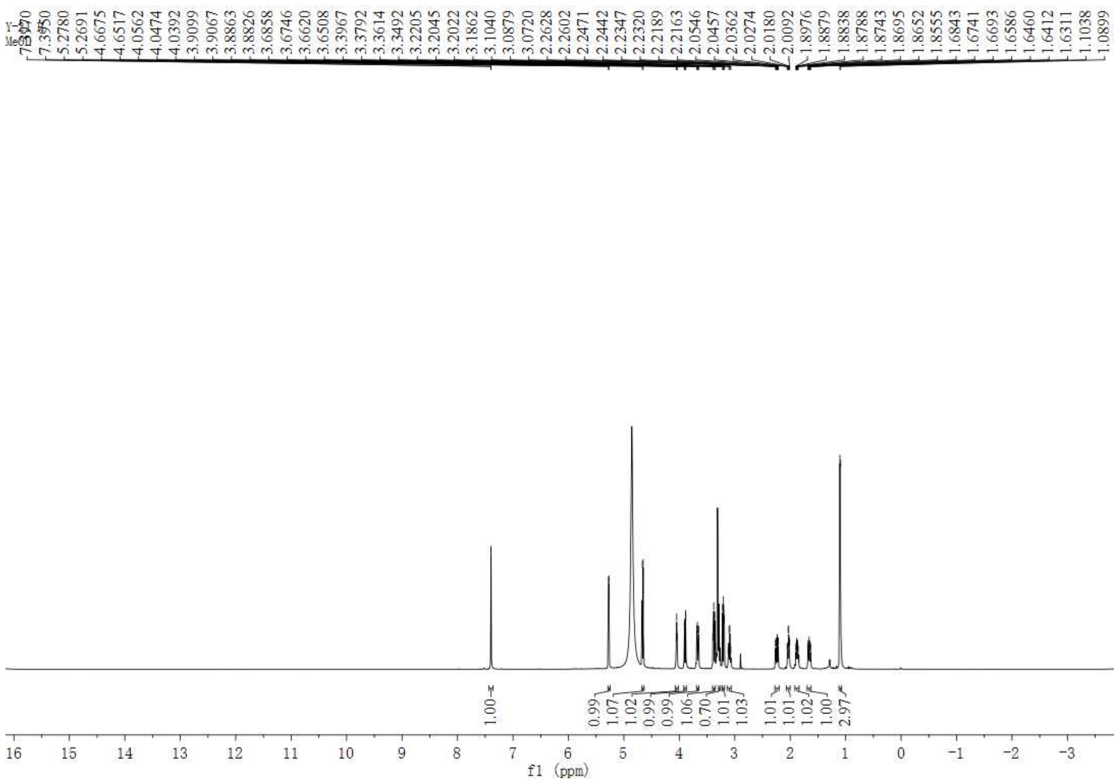

Figure S45. ^{1}H NMR spectrum of compound **5** (500 MHz, MeOD)

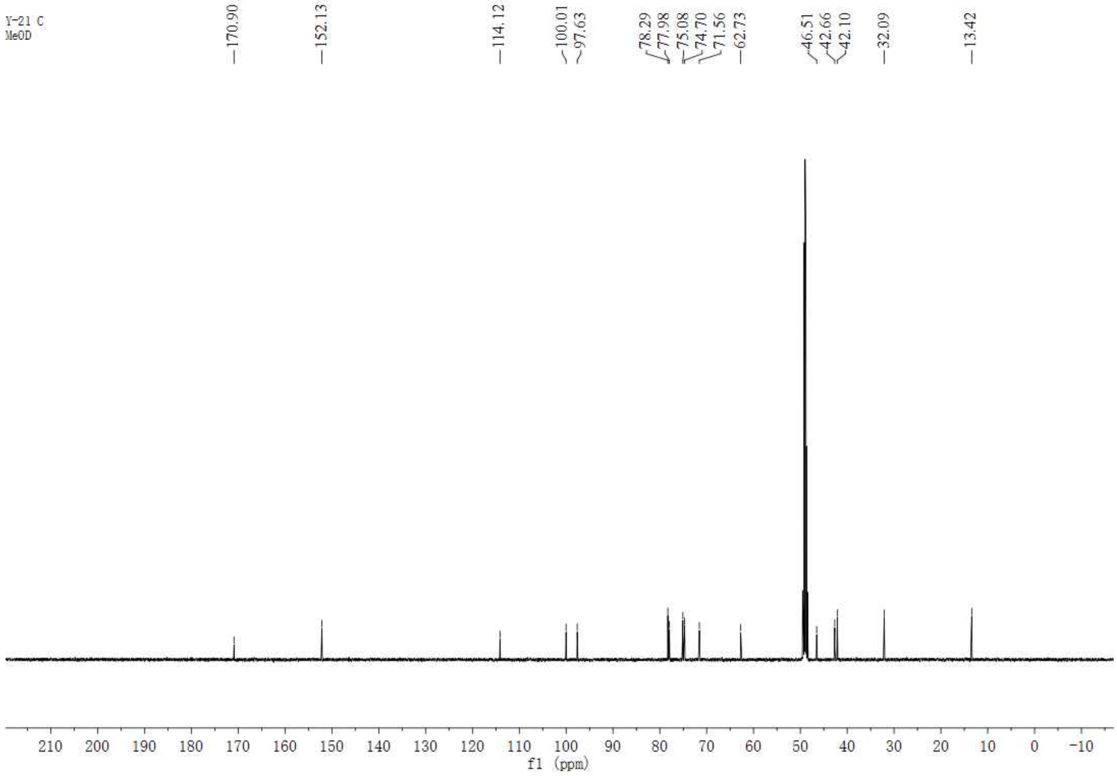

Figure S46. ^{13}C NMR spectrum of compound **5** (125 MHz, MeOD)

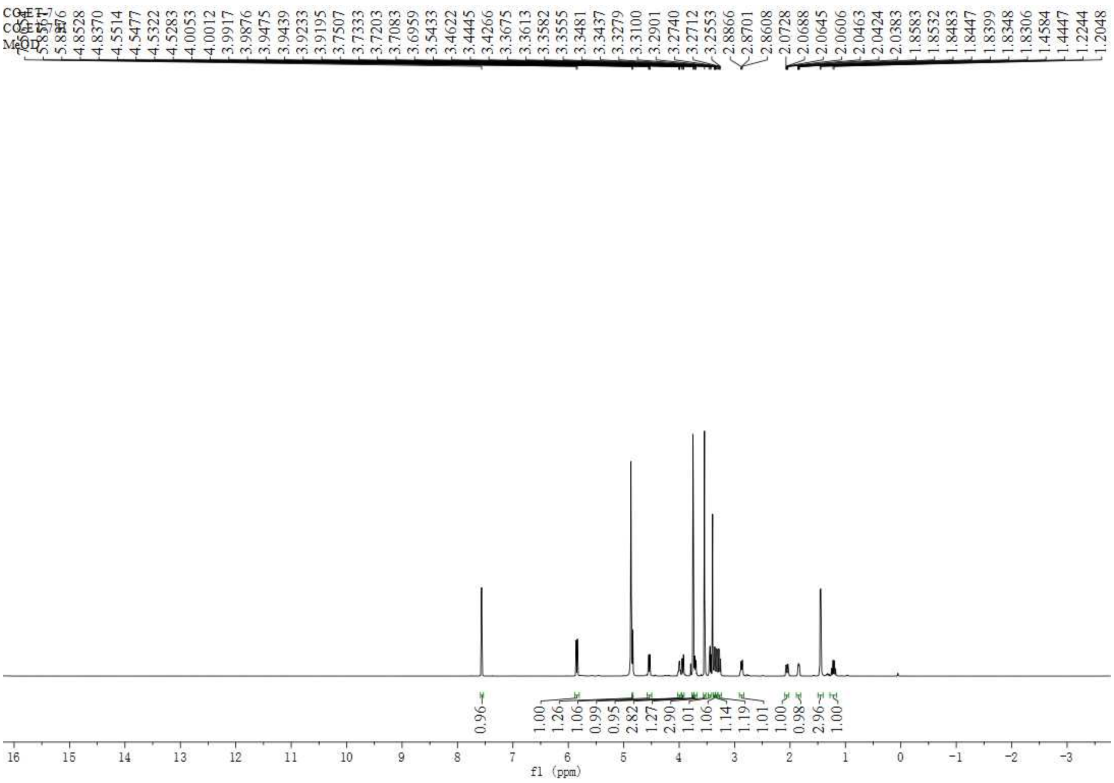

Figure S47. ^{1}H NMR spectrum of compound **6** (500 MHz, MeOD)

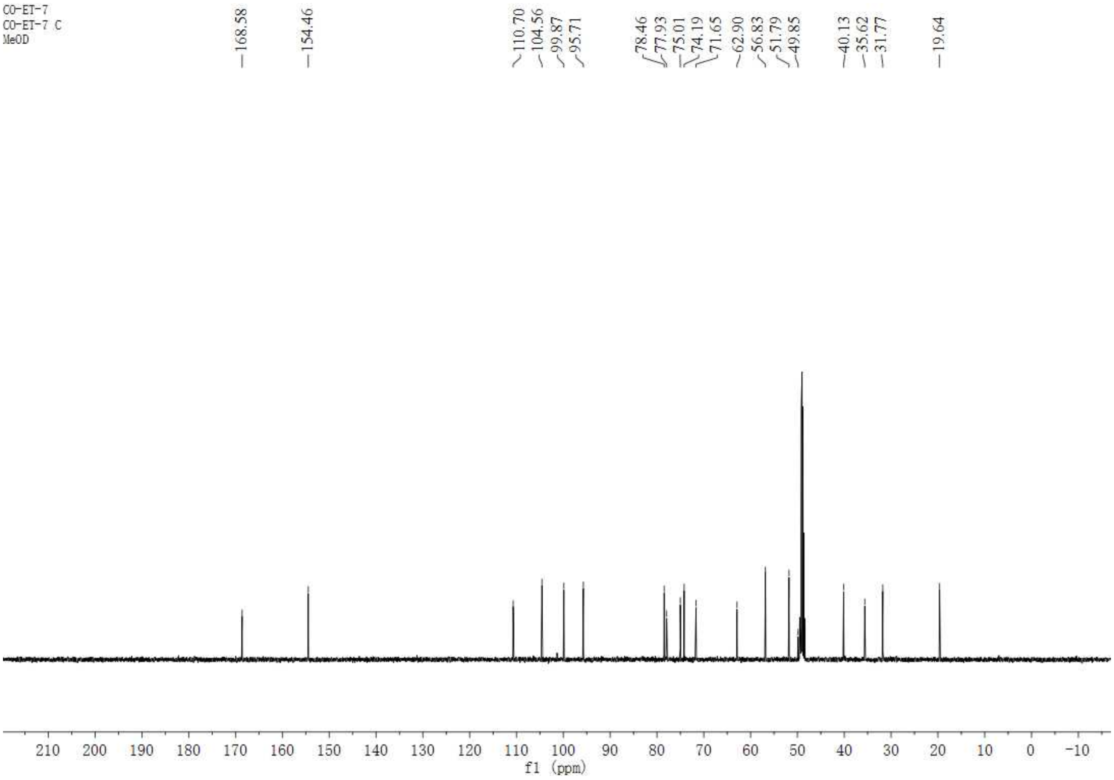

Figure S48. ^{13}C NMR spectrum of compound **6** (125 MHz, MeOD)

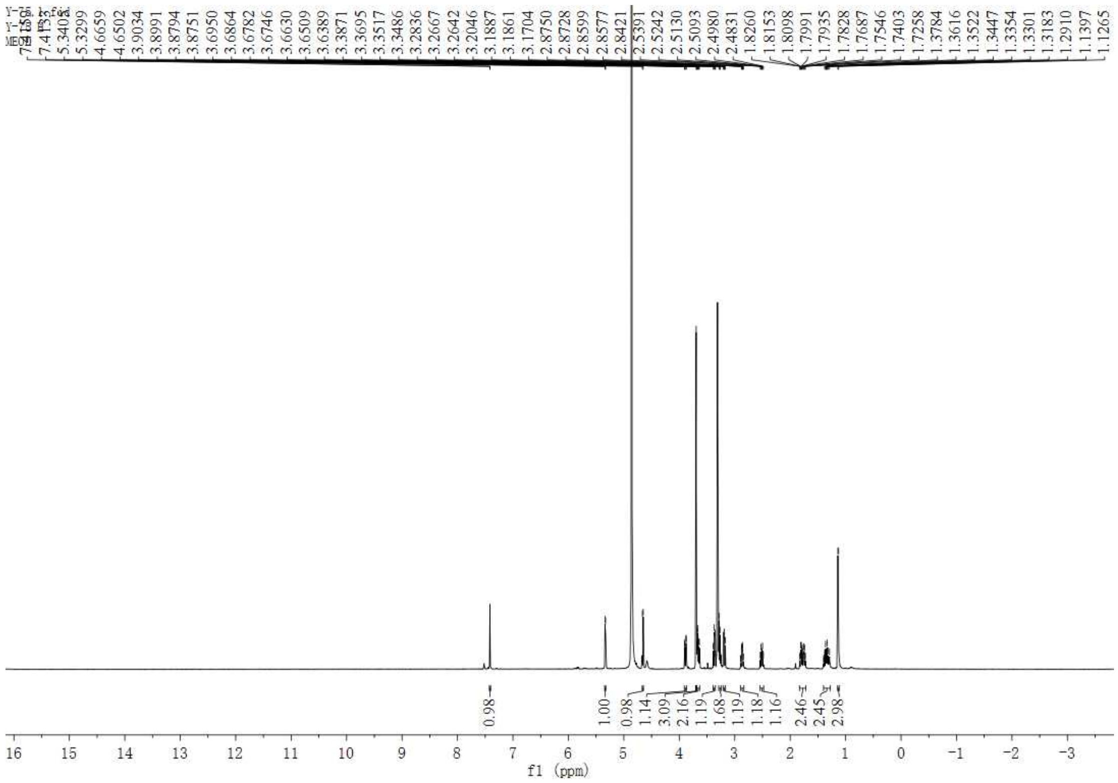

Figure S49. ^{1}H NMR spectrum of compound **7** (500 MHz, MeOD)

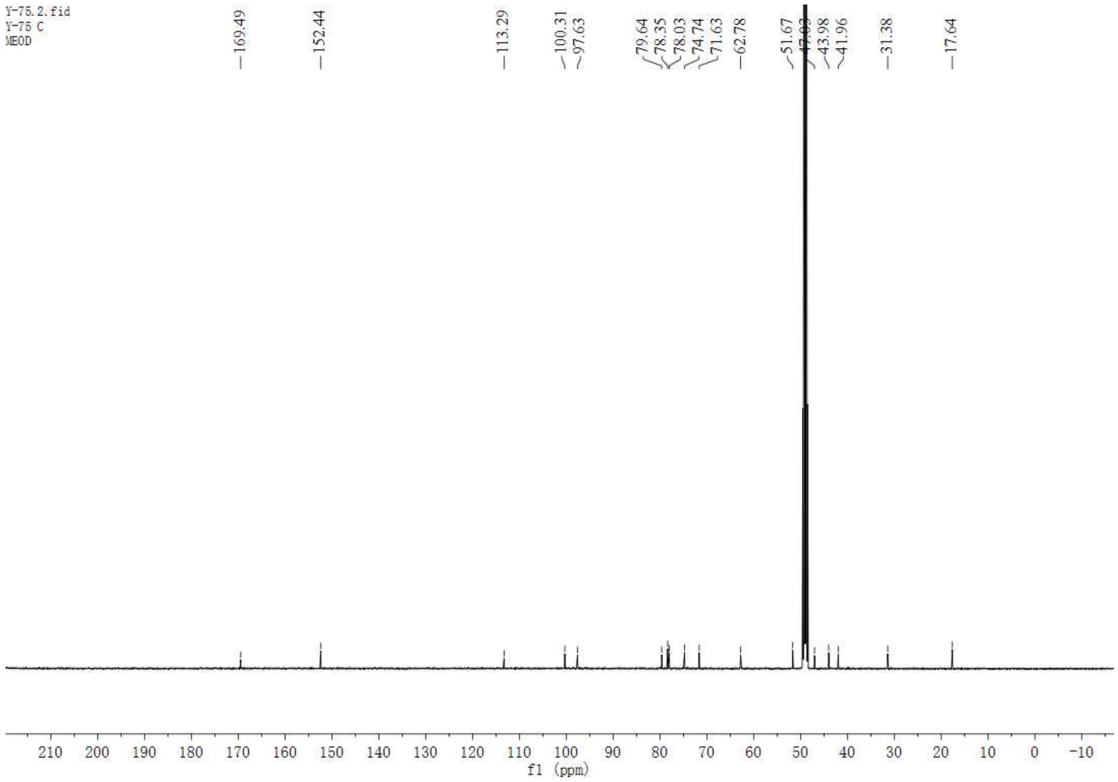

Figure S50. ^{13}C NMR spectrum of compound **7** (125 MHz, MeOD)

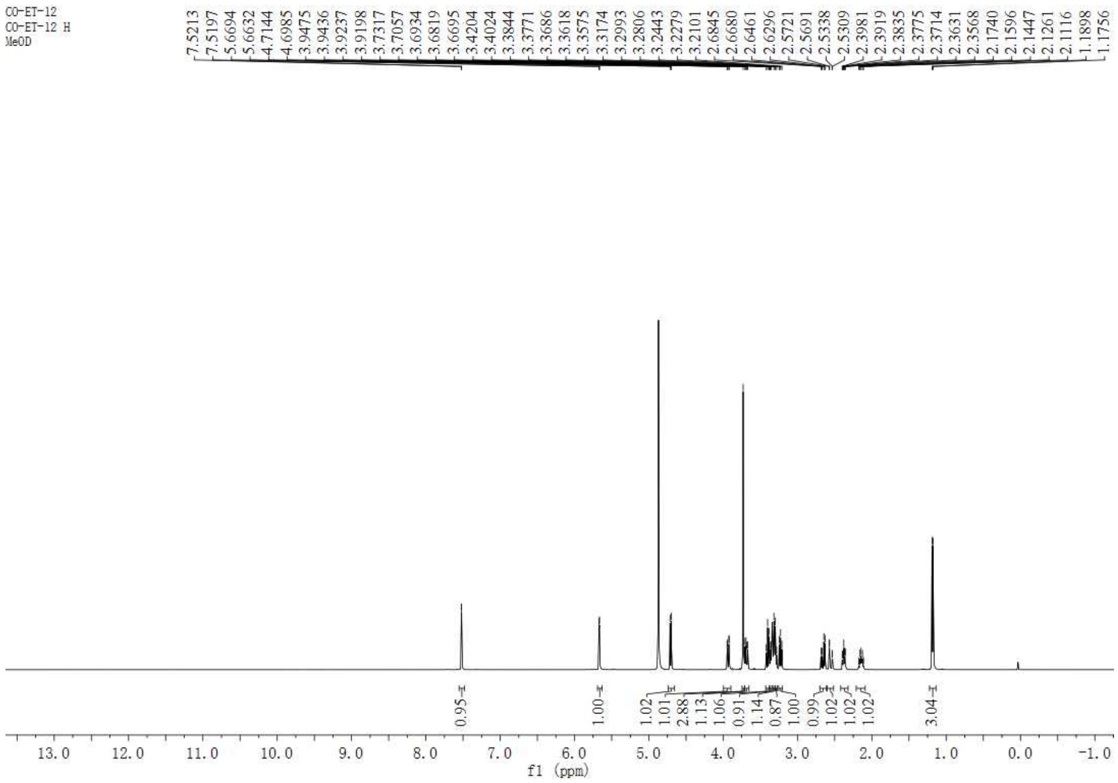

Figure S51. ^{1}H NMR spectrum of compound **8** (500 MHz, MeOD)

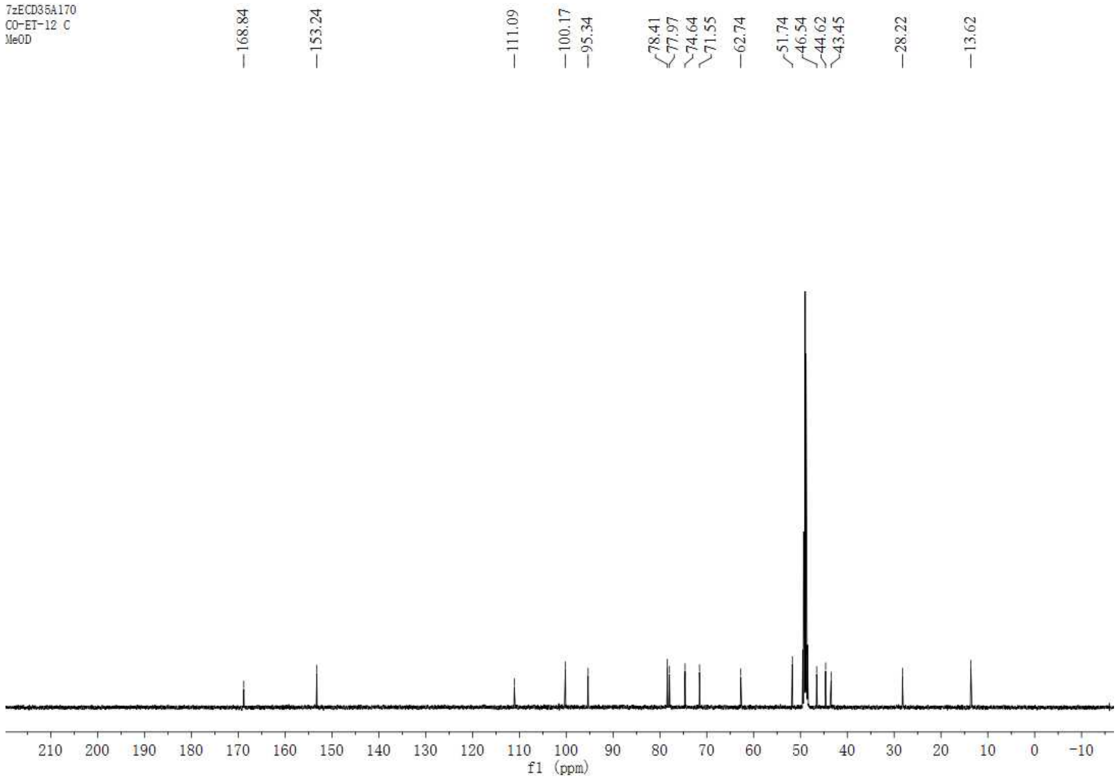

Figure S52. ^{13}C NMR spectrum of compound **8** (125 MHz, MeOD)

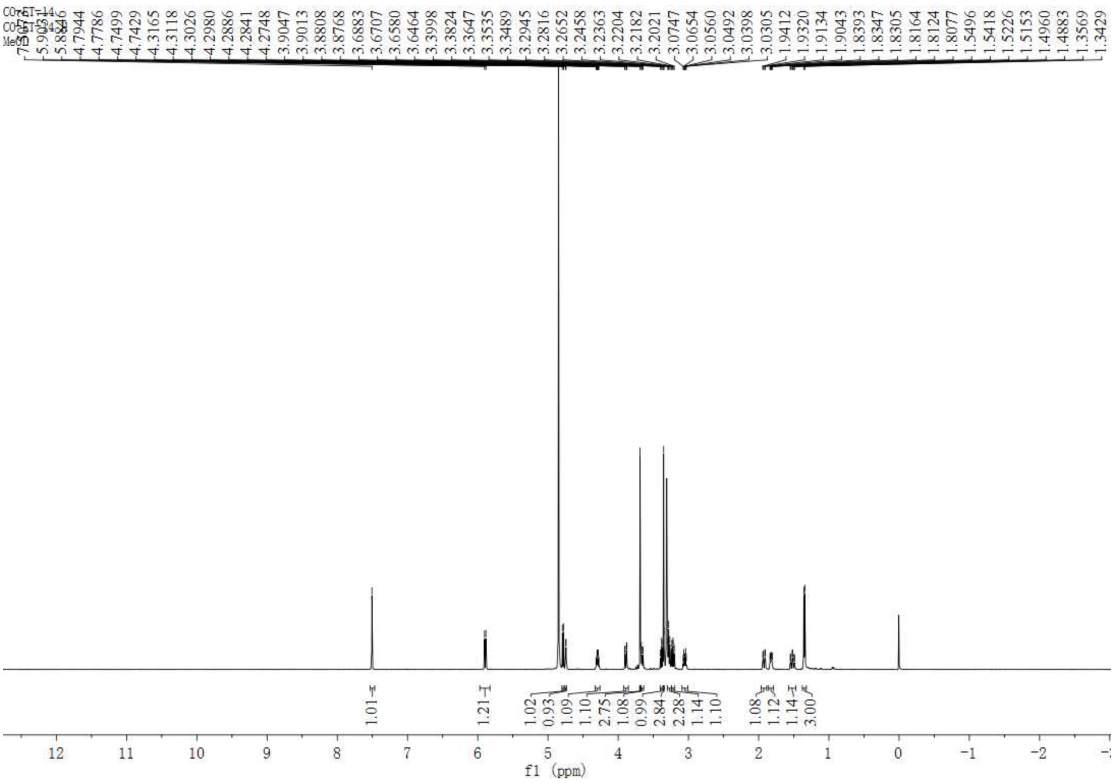

Figure S53. ^{1}H NMR spectrum of compound **9** (500 MHz, MeOD)

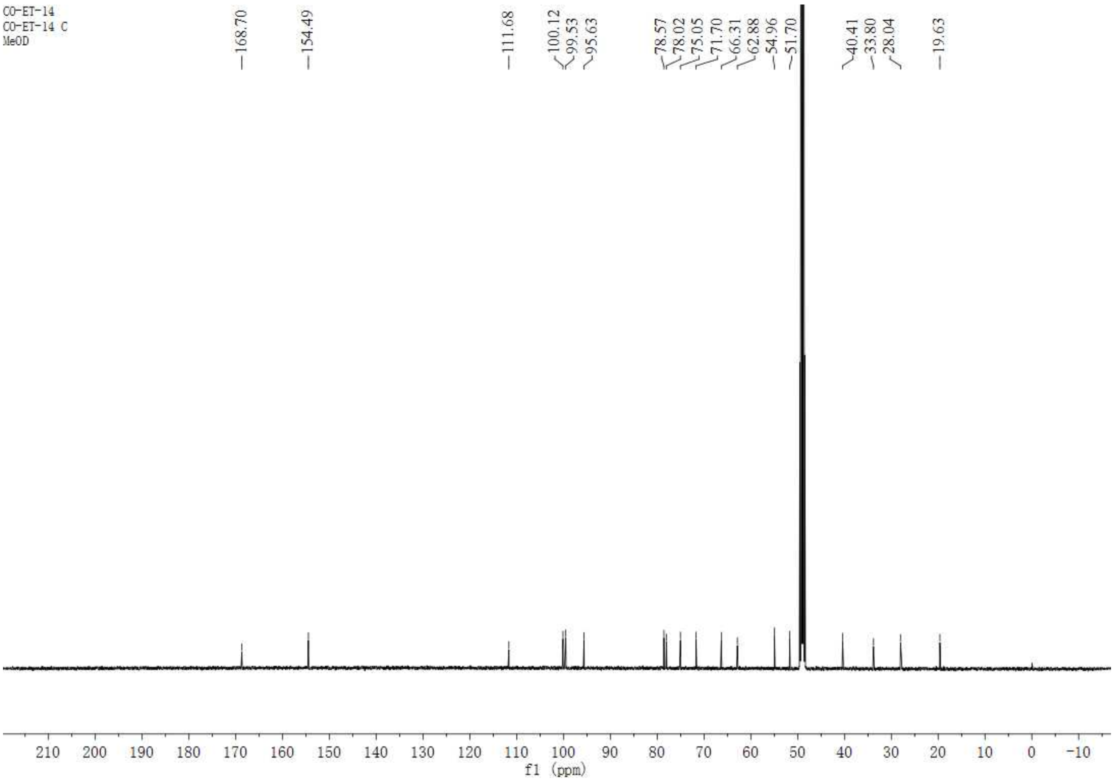

Figure S54. ^{13}C NMR spectrum of compound **9** (125 MHz, MeOD)

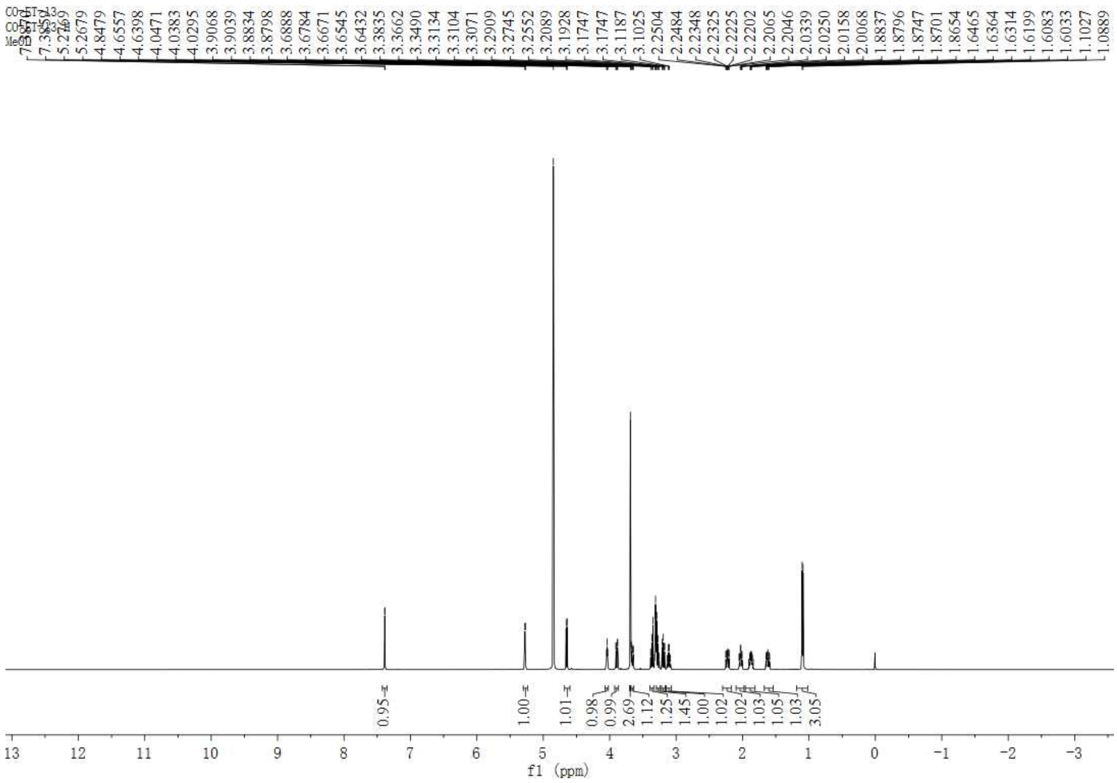

Figure S55. ^{1}H NMR spectrum of compound **10** (500 MHz, MeOD)

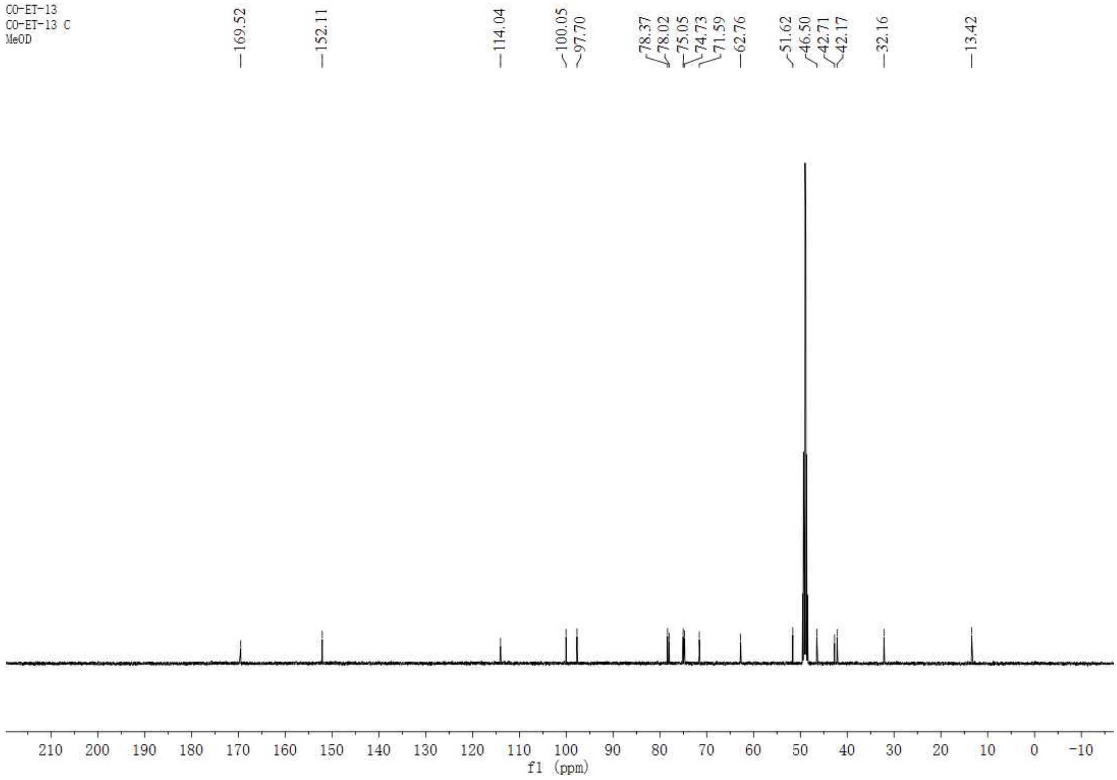

Figure S56. ^{13}C NMR spectrum of compound **10** (125 MHz, MeOD)

Table S1. Effect of compounds **1**–**10** on the relative glucose consumption in insulin-induced HepG2 cells ($\bar{x}$ ± s, n = 4).

Group	Dose (μM)	The relative glucose consumption (mM/OD)
Control		$1.430 \pm 0.044^{**}$
Model		$0.480 \pm 0.121^{\#\#}$
Rosiglitazone	10	$1.330 \pm 0.090^{**}$
**1**	10	$1.290 \pm 0.330^{**}$
**2**	10	0.780 ± 0.070
**3**	10	$0.980 \pm 0.120^{*}$
**4**	10	0.720 ± 0.170
**5**	10	0.910 ± 0.081
**6**	10	0.850 ± 0.228
**7**	10	$1.020 \pm 0.099^{*}$
**8**	10	0.656 ± 0.116
**9**	10	0.245 ± 0.050
**10**	10	0.546 ± 0.174

$^{\#}P < 0.05$ or $^{\#\#}P < 0.01$, versus control group; $^{*}P < 0.05$ or $^{**}P < 0.01$, versus insulin group.

Table S2. Effect of compounds **1**, **3**, and **7** on the relative glucose consumption in insulin-induced HepG2 cells ($\bar{x}$ ± s, n = 6).

Group	Dose (μM)	The relative glucose consumption (mM/OD)
Control		2.731 ± 0.994
Model		0.558 ± 0.281
Rosiglitazone	0.625	1.261 ± 0.322
	1.25	1.603 ± 0.613
	2.5	1.719 ± 0.548
	5	1.771 ± 0.103
	10	2.280 ± 0.554
	20	2.133 ± 0.436
	30	2.106 ± 0.528
**1**	0.625	0.374 ± 0.146
	1.25	0.972 ± 0.479
	2.5	1.053 ± 0.280
	5	1.656 ± 0.228
	10	1.712 ± 0.572
	20	1.767 ± 0.401
	30	3.342 ± 1.557
**3**	0.625	0.738 ± 0.300
	1.25	0.858 ± 0.510
	2.5	1.428 ± 0.134
	5	1.508 ± 0.122
	10	1.585 ± 0.165
	20	1.715 ± 0.470
	30	1.891 ± 0.434
**7**	0.625	0.356 ± 0.068
	1.25	1.530 ± 0.834
	2.5	1.768 ± 0.352
	5	2.178 ± 1.167
	10	1.788 ± 0.628
	20	1.758 ± 0.514
	30	1.791 ± 0.210
